# Hypomorphic ASGR1 modulates lipid homeostasis via INSIG1-mediated SREBP signaling suppression

**DOI:** 10.1172/jci.insight.147038

**Published:** 2021-10-08

**Authors:** Yingying Xu, Jiawang Tao, Xiaorui Yu, Yuhang Wu, Yan Chen, Kai You, Jiaye Zhang, Anteneh Getachew, Tingcai Pan, Yuanqi Zhuang, Fang Yuan, Fan Yang, Xianhua Lin, Yin-xiong Li

**Affiliations:** 1 Center for Health Research, Guangdong Provincial Key Laboratory of Biocomputing, Guangzhou Institutes of Biomedicine and Health, Chinese Academy of Sciences, Guangzhou, China.; 2 University of Chinese Academy of Sciences, Beijing, China.; 3 School of Life Sciences, University of Science and Technology of China, Hefei, China.; 4 Guangdong Provincial Key Laboratory of Stem Cell and Regenerative Medicine, Guangzhou, China.; 5 Bioland Laboratory (Guangzhou Regenerative Medicine and Health Guangdong Laboratory), Guangzhou, China.

**Keywords:** Metabolism, Atherosclerosis, Cholesterol, Lipoproteins

## Abstract

A population genetic study identified that the asialoglycoprotein receptor 1 (*ASGR1*) mutation carriers had substantially lower non–HDL-cholesterol (non–HDL-c) levels and reduced risks of cardiovascular diseases. However, the mechanism behind this phenomenon remained unclear. Here, we established *Asgr1*-knockout mice that represented a plasma lipid profile with significantly lower non–HDL-c and triglyceride (TG) caused by decreased secretion and increased uptake of VLDL/LDL. These 2 phenotypes were linked with the decreased expression of microsomal triglyceride transfer protein and proprotein convertase subtilisin/kexin type 9, 2 key targeted genes of sterol regulatory element–binding proteins (SREBPs). Furthermore, there were fewer nuclear SREBPs (nSREBPs) on account of more SREBPs being trapped in endoplasmic reticulum, which was caused by an increased expression of insulin-induced gene 1 (INSIG1), an anchor of SREBPs. Overexpression and gene knockdown interventions, in different models, were conducted to rescue the ASGR1-deficient phenotypes, and we found that INSIG1 knockdown independently reversed the ASGR1-mutated phenotypes with increased serum total cholesterol, LDL-c, TG, and liver cholesterol content accompanied by restored SREBP signaling. ASGR1 rescue experiments reduced INSIG1 and restored the SREBP network defect as manifested by improved apolipoprotein B secretion and reduced LDL uptake. Our observation demonstrated that increased INSIG1 is a critical factor responsible for ASGR1 deficiency–associated lipid profile changes and nSREBP suppression. This finding of an ASGR1/INSIG1/SREBP axis regulating lipid hemostasis may provide multiple potential targets for lipid-lowering drug development.

## Graphical abstract

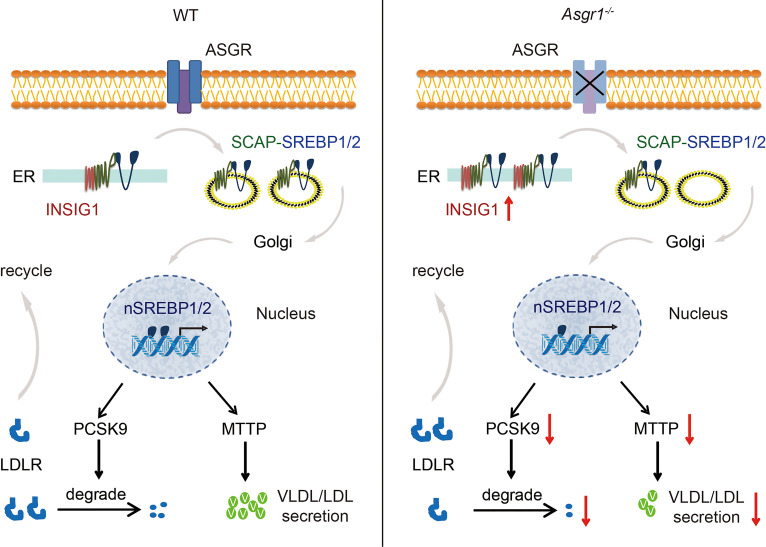

## Introduction

Dyslipidemia is a major risk factor for the development of metabolic diseases, including fatty liver, type 2 diabetes mellitus, cardiovascular disease (CVD), and stroke. The most used lipid-lowering drugs are statins (HMG-CoA reductase inhibitors) that have been found to reduce CVD and mortality but with therapeutic limitations (existing statin-insensitive patients) and potential side effects (rhabdomyolysis and hepatotoxicity) (1). Thus, new therapeutic strategies for metabolic diseases are necessary. Elevated non–HDL-cholesterol (non–HDL-c) content is believed to be a leading cause of CVD (2). A human population genetic study provided evidence that variant asialoglycoprotein receptor 1 (*ASGR1*) is associated with lower non–HDL-c content and reduced risk of coronary artery disease (CAD), indicating a potential target for CVD prevention and treatment (3).

ASGR1 is the major subunit of ASGR, a transmembrane glycoprotein predominantly expressed on the sinusoidal surface of the hepatocytes in the liver (4). ASGR functions as a C-type lectin mediating endocytosis and degradation of desialylated proteins in circulation (5). It plays a pivotal role in a variety of pathophysiologic processes, such as removal of desialylated platelets (6, 7), suppression of hepatocellular carcinoma metastasis (8), elimination of activated lymphocytes (9), and serving as the gate for hepatotropic viruses (10, 11). Notably, ASGR is also postulated to account for lipoprotein clearance. Lipoprotein (a) and chylomicron remnant have emerged as likely ligands for ASGR (12, 13). With support of the population genetic study data, ASGR1 is emerging as an attractive protein for dissecting the regulatory network of lipid homeostasis and a potential target for lipid-lowering drug development.

In mammals, sterol regulatory element–binding proteins (SREBPs) play a vital role in cholesterol and lipid homeostasis. SREBPs have 4 isoforms, designated SREBP1a, SREBP1b, SREBP1c, and SREBP2 (14, 15). SREBPs function as transcriptional factors via negative feedback control to regulate lipogenesis and uptake and biosynthesis of cholesterol. SREBP1 is responsible for fatty acid synthesis and energy storage, while SREBP2 is responsible for cholesterol regulation (16–19). Activation of SREBPs is tightly regulated by intracellular sterol condition. In the presence of sterols, SREBPs are anchored in the endoplasmic reticulum (ER) with SREBP-cleavage-activating protein (SCAP) and insulin-induced gene 1/2 (INSIG1/2). In the absence of sterols, the SCAP/SREBP complex dissociates from INSIG1/2 and is packaged into coatomer protein subunit beta 1 vesicles to travel to the Golgi apparatus, where SREBPs undergo sequential proteolytic cleavages by site 1 protease and site 2 protease to release the NH2-terminal domain. Then, the active nuclear SREBPs (nSREBPs) enter the nucleus and bind the sterol response element (SRE) to regulate downstream genes associated with lipogenesis and metabolism (19, 20). In the process, SCAP and INSIG1/2 are the core sterol sensors that control the SREBP pathway. Except for sterol, the space configuration and posttranslational modifications such as phosphorylation and glycosylation also affect functions of these proteins (21–24). However, the association of ASGR1 with the SREBP pathway remains unknown, and it needs further investigation to improve our understanding of lipid homeostasis.

Here, we study the functions of ASGR1 in the regulation of systemic lipid metabolism. In line with human genetic findings, ASGR1 deficiency in mice reduced cholesterol and triglyceride (TG) contents. Mechanistic studies revealed that ASGR1 deficiency promoted SREBPs’ retention in ER through upregulating INSIG1 and inhibiting SREBPs’ activation. These findings suggest ASGR1 inhibition can be a potential strategy to reduce plasma lipid content.

## Results

### ASGR1-knockout mice represent a low lipid profile.

**Figure 1:**
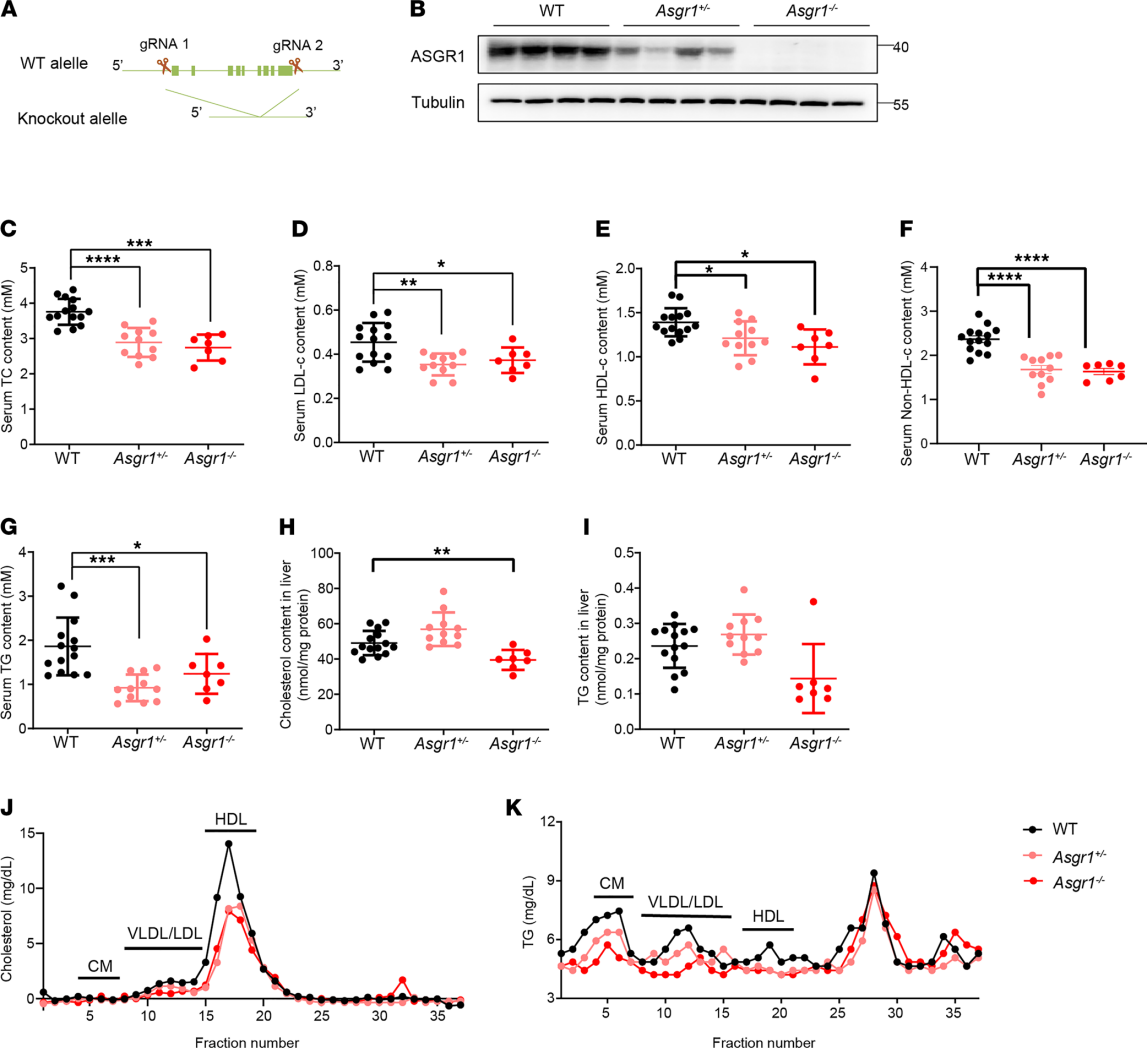
ASGR1-knockout mice represent a low lipid profile. (**A**) Illustration of CRISPR/Cas9 targeting strategy for *Asgr1* deletion in mice. Two single-guide RNAs were applied for gene edition. (**B**) ASGR1 protein contents in the livers of WT, *Asgr1*^+/–^, and *Asgr1*^–/–^ mice were analyzed by Western blots, *n* = 4. Biochemical index including (**C**–**G**) total cholesterol (TC), LDL-c, HDL-c, non–HDL-c, and triglyceride (TG) contents in serum of mice fasted overnight were analyzed (WT, *n* = 14; *Asgr1*^+/–^, *n* = 11; *Asgr1*^–/–^, *n* = 7). In parallel (**H** and **I**), cholesterol and TG contents in liver tissues were analyzed (WT, *n* = 14; *Asgr1*^+/–^, *n* = 11; *Asgr1*^–/–^, *n* = 7). Lipid profiles were analyzed by fast protein liquid chromatography (FPLC) for pooled serum from mice fasted overnight (*n* = 7). (**J**) Cholesterol and (**K**) TG content in each fraction eluted from FPLC (WT, *n* = 7; *Asgr1*^+/–^, *n* = 7; *Asgr1*^–/–^, *n* = 7). All data are shown as the means ± SD. In all Western blots, the numbers on the right are molecular weights of protein markers (kDa). *P < 0.05, **P < 0.01, ***P < 0.001, ****P < 0.0001 as compared with the indicated WT by 1-way ANOVA.

To investigate the physiological functions of ASGR1, we first generated *Asgr1*-knockout mice with CRISPR/Cas9 gene editing, and the obtained knockout lines were confirmed by Western blotting (Figure 1A and Supplemental Figure 1, A and B; supplemental material available online with this article; https://doi.org/10.1172/jci.insight.147038DS1). ASGR1 was highly expressed in liver tissues of WT mice but was substantially decreased or undetectable in *Asgr1*^+/−^ or *Asgr1*^−/−^ mice, respectively (Figure 1B). The body weight of mice and hepatic histology showed no significant difference among mice with different genotypes (Supplemental Figure 1, C and E). Notably, biochemical analysis of the lipid profile indicated that the whole spectrum of lipids in serum and liver tissues were decreased in ASGR1-deficient male mice (Figure 1, C–I). In detail, the serum TC level showed significant changes among groups, which were 3.8 (± 0.50) mM, 2.9 (± 0.43) mM, and 2.7 (± 0.37) mM in WT, *Asgr1*^+/−^, and *Asgr1*^−/−^ mice, respectively, followed by the reduced level of LDL-c, HDL-c, and TG in similar trends. Considering that the reported variant *ASGR1* was associated with reduced non–HDL-c, we calculated non–HDL-c level by subtracting HDL-c content from TC content. The non–HDL-c content of the WT, *Asgr1*^+/−^, and *Asgr1*^−/−^ mice was 2.3 (± 0.45) mM, 1.7 (± 0.3) mM, and 1.63 (± 0.19) mM, respectively (Figure 1F). To dissect out lipoprotein class–linked cholesterol and TG changes, the FPLC assay was performed. Corresponding fractions (chylomicron, VLDL/LDL, and HDL) were sequentially collected and measured for the cholesterol and TG concentrations. Consistent with the trends and changes of biochemical analysis, the FPLC assay confirmed that the content of lipids carried by lipoproteins was decreased (Figure 1, J and K).

To detect whether there is a sex difference in ASGR1 physiological functions, we repeated the lipid profile analyses in female mice. Consistent with male mice, the serum TC, LDL-c, HDL-c, and non–HDL-c levels were decreased in ASGR1-deficient female mice, with the exception of TG (Supplemental Figure 2, A–E). Therefore, we used male mice in the following experiments to avoid potential hormonal effects. Such lipid profiling analyses in vivo indicated that ASGR1 was involved in the regulatory network of lipid homeostasis.

### VLDL/LDL secretion is reduced, accompanied by less microsomal triglyceride transfer protein in ASGR1-deficient mice.

**Figure 2:**
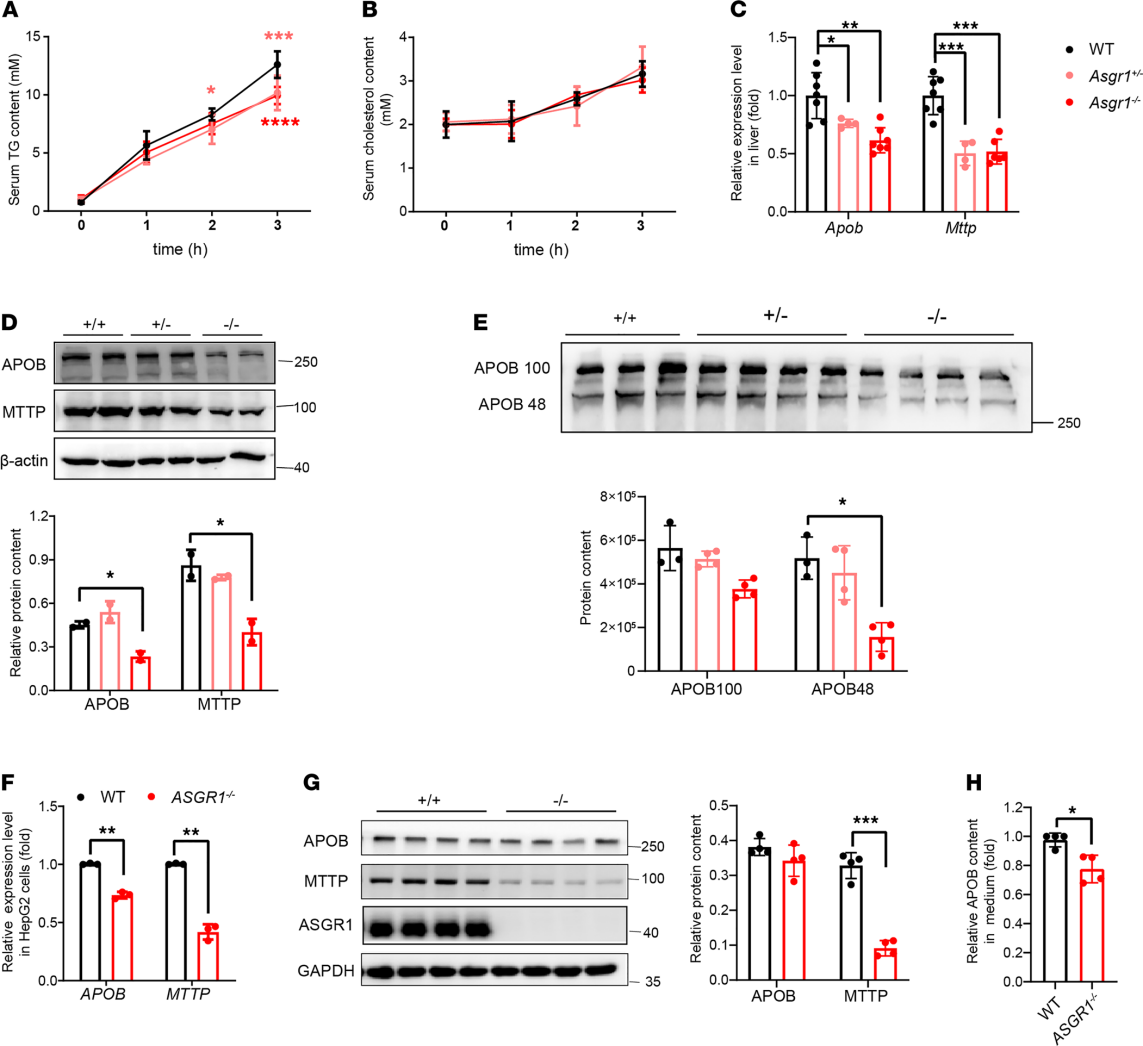
VLDL/LDL secretion is reduced accompanied with less microsomal triglyceride transfer protein in ASGR1-deficient mice. Hepatic lipid secretion assays were conducted on mice with different genotypes. (**A**) TG and (**B**) cholesterol content were measured at 0, 1, 2, and 3 hours after i.p. injection with tyloxapol, *n* = 5. (**C**) The relative mRNA expression level of secretion-related genes, *Apob* and microsomal triglyceride transfer protein (*Mttp*) were analyzed by real-time PCR (RT-PCR) in liver tissues (WT, *n* = 7; *Asgr1*^+/–^, *n* = 4; *Asgr1*^–/–^, *n* = 7). (**D**) The corresponding proteins, APOB and MTTP, were analyzed by Western blots in liver tissues. Representative images of the immunoblotting and gray intensity of each band relative to β-actin was shown, *n* = 3. (**E**) Secreted APOB content in mouse serum was analyzed by Western blots (WT, *n* = 3; *Asgr1*^+/–^, *n* = 4; *Asgr1*^–/–^, *n* = 4). Parallel analyses were conducted on HepG2 cell lines. (**F**) The mRNA expression level of *APOB* and *MTTP* was analyzed by RT-PCR (*n* = 4), and (**G**) protein content of APOB and MTTP was analyzed by Western blots, *n* = 4. (**H**) ELISA comparison of secreted APOB content between WT and *ASGR1*^–/–^, *n* = 4. All data are shown as the means ± SD. *P < 0.05, **P < 0.01, ***P < 0.001, ****P < 0.0001 as compared with the indicated WT by 1-way ANOVA among 3 groups. Statistical significance between 2 groups was assessed with an unpaired, 2-tailed *t* test.

Dietary intake and absorption as well as lipid secretion could affect the serum lipid levels. To determine the factor responsible for the low lipid profile in ASGR1-deficient mice, the amount of food intake per day for mice was measured, and the results suggested that mice with different genotypes took in a similar amount of food (Supplemental Figure 1D). Then an oral lipid tolerance test was conducted to measure lipid absorption ability among the mice. By gavage of olive oil to mice, after 4 hours, we found that the serum TG and cholesterol content were similar among WT and ASGR1-deficient mice, indicating that ASGR1 deficiency did not affect dietary fat absorption (Supplemental Figure 3, A and B). Next, to determine whether deficiency of ASGR1 can influence lipid secretion, we measured serum lipid level after administration of lipoprotein lipase inhibitor tyloxapol and found serum TG levels of ASGR1-deficient mice were comparatively less than those of WT mice. The most significant difference was observed at 3 hours after intravenous injection (*Asgr1*^+/−^, decreased by 19%; *Asgr1*^−/−^, decreased by 21%; Figure 2, A and B).

Within this short experimental period, the measured secretion of TC did not show a significant difference among the groups of ASGR1-knockout and WT mice. However, the deep analysis of apolipoprotein B (APOB, the core factor of VLDL/LDL) with FPLC confirmed that the APOB content was reduced in fractions containing CM and VLDL/LDL particles in serum of the *Asgr1*^−/−^ mice, which was consistent with the observed changes of the serum lipoprotein profile (Supplemental Figure 3C and Figure 1). Western blot analyses also revealed less APOB in the liver and serum of ASGR1-deficient mice. Particularly, in the null *Asgr1*-nullmice, the serum contents of APOB100 and APOB48 were decreased by 40% and 60% (Figure 2, D and E). The decrease was paralleled with the similar trend of reduction of MTTP (the key protein for LDL assembly and secretion). The reduction of APOB and MTTP occurred not only on the protein level but also on the transcriptional level; the mRNA expression of *Mttp* and *Apob* was significantly decreased in null *Asgr1*-null mice compared with that of WT counterparts (Figure 2C).

Furthermore, to verify the effect of ASGR1 on LDL secretion, *ASGR1* was knocked out in HepG2 cells. The secreted APOB in the supernatant was significantly decreased in *ASGR1*-null HepG2 cells, corresponding to the animal results (Figure 2H). Similarly, MTTP was decreased significantly at both mRNA and protein levels in ASGR1-deficient cells (Figure 2, F and G). In summary, ASGR1 deficiency led to a defect in LDL secretion that might be caused by less MTTP and APOB for the construction of the LDL complex and its secretion from hepatocytes.

### The LDL uptake rate increases in ASGR1-deficient hepatocytes linked with less proprotein convertase subtilisin/kexin type 9.

**Figure 3:**
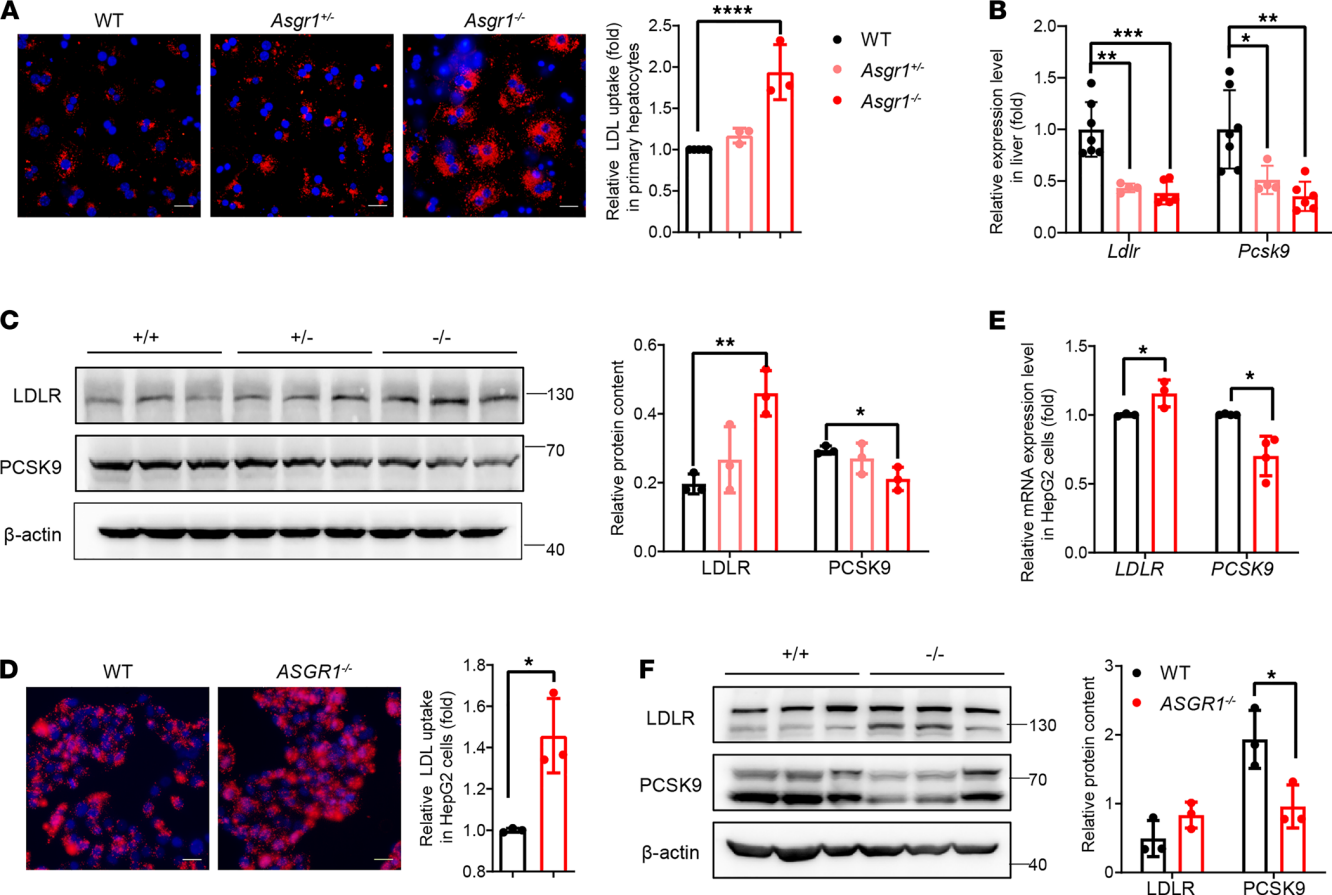
The LDL uptake rate increased in ASGR1-deficient hepatocytes linked with less proprotein convertase subtilisin/kexin type 9. The LDL uptake assays were conducted on primary hepatocytes with different genotypes. (**A**) Representative images of LDL-dil (red) accumulation and nuclear staining (blue) with Hoechst 33258 (scale bar: 20 μm) with florescence intensity quantitative analysis (right). Each point represents 1 of the experimental replicates, *n* = 3. (**B**) The mRNA expression level of LDL receptor (*Ldlr*) and proprotein convertase subtilisin/kexin type 9 (*Pcsk9*) was analyzed by RT-PCR in liver tissues. WT, *n* = 7; *Asgr1*^+/–^, *n* = 4; *Asgr1*^–/–^, *n* = 6. (**C**) The corresponding proteins, LDLR and PCSK9, were analyzed by Western blots in liver tissues, *n* = 3. Gray intensity of the band was shown on the right. Parallel analyses were conducted on HepG2 cell lines. (**D**) Representative images of LDL-dil (red) accumulation and nuclear staining (blue) with Hoechst 33258 (scale bar: 20 μm) with fluorescence intensity quantitative analysis (right). Each point represents 1 of the experimental replicates, *n* = 3. (**E**) The mRNA expression level of *LDLR* and *PCSK9* in cells, *n* = 3. (**F**) The corresponding proteins, LDLR and PCSK9, were analyzed by Western blots in cells. Gray intensity of the band was shown on the right, *n* = 3. All data are shown as the means ± SD. *P < 0.05, **P < 0.01, ***P < 0.001, ****P < 0.0001 as compared with the indicated WT by 1-way ANOVA among 3 groups. Statistical significance between 2 groups was assessed with an unpaired, 2-tailed *t* test.

The uptake of LDL-c by the liver is the predominant way to clear it from plasma. To investigate whether hepatic LDL uptake ability could be affected by ASGR1, the LDL uptake assay was conducted on the primary hepatocytes isolated from mice and HepG2 cells without or with ASGR knockout (ASGR1^–/–^). Hence, dil-labeled LDL was incubated with hepatic cells for 4 hours after starvation of the cells overnight. In ASGR1-deficient hepatic cells, the fluorescence intensities of such internalized LDL-dil particles were markedly higher than those of the WT cells. In detail, the uptake rates increased by 20% and 90% in *Asgr1*^+/–^ and *Asgr1*^–/–^ primary hepatocytes, respectively, compared with the WT counterparts. Likewise, the LDL uptake rate was increased by 45% in *ASGR1*^–/–^ HepG2 cells compared with control WT cells (Figure 3, A and D). For further validation, we established ASGR1-knockout human hepatocyte-like cells differentiated from embryonic stem cell line H1 (hu-HLC-*ASGR1*^–/–^) (Supplemental Figure 4, A and B) and measured LDL uptake rate. Consequently, increased LDL uptake was observed in the hu-HLC-*ASGR1*^–/–^ cells by 50% (Supplemental Figure 4C). In addition, the LDLR expression level was determined both at mRNA and protein levels. The *LDLR* mRNA expression declined in ASGR1-deficient livers, and a slight increase was observed in HepG2 cells. In contrast to the mRNA expression pattern, increased LDLR protein was observed in all cases of ASGR1-deficient mice and HepG2 cells (Figure 3, C and F). Furthermore, PCSK9, the mediator for LDLR degradation (25), was found to be significantly downregulated both on mRNA and on protein levels in ASGR1-deficient conditions, which contributed to stability of LDLR (Figure 3, B, C, E, and F). Upon overexpression of PCSK9 in HepG2-ASGR1^–/–^ cells and WT HepG2 cells, respectively, the LDLR protein content decreased in both cell lines, leading to no substantial difference of the LDLR between HepG2-ASGR1^–/–^ and WT cells (Supplemental Figure 4D). Collectively, the increased LDL uptake under ASGR1-deficient conditions might be due to the low PCSK9 level leading to more stable LDLR on hepatocytes.

### Attenuated metabolic gene program in Asgr1^–/–^ mouse livers.

**Figure 4:**
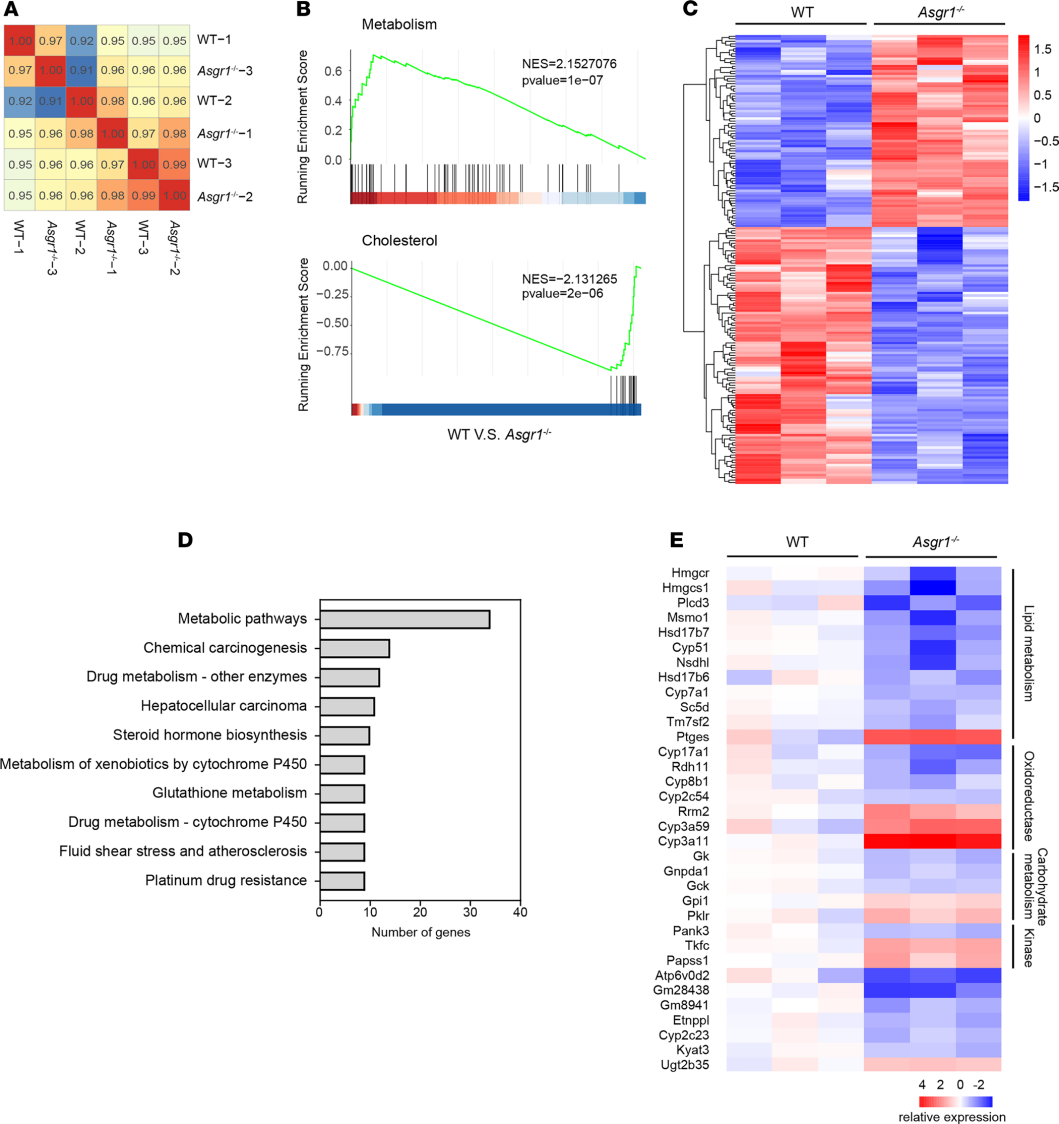
Attenuated metabolic gene program in livers of *Asgr1*^–/–^ mice. Liver tissues of WT and *Asgr1*^–/–^ mice were collected for RNA-Seq and analyzed with WT as control, *n* = 3. (**A**) Heatmap shows hierarchical clustering of Pearson’s correlation coefficient for all liver samples based on RNA-Seq profiles. Score of 1 (red) denotes perfect correlation. (**B**) GSEAs of gene sets for metabolism (top) and cholesterol homeostasis (bottom). NES, normalized enrichment score. (**C**) Heatmap shows hierarchical clustering of differentially expressed genes in *Asgr1*^–/–^ mice. Values are column-scaled to show expression level. (**D**) Kyoto Encyclopedia of Genes and Genomes analyses of differentially expressed genes. Values are row-scaled to show number of genes involved in the relevant pathway. (**E**) Heatmap shows hierarchical clustering of differentially expressed genes involved in the metabolic pathway. Values are row-scaled to show relative expression level.

To investigate underlying mechanisms, RNA-Seq on liver tissues of WT and *Asgr1*^–/–^ mice was performed. The majority of transcriptomes of *Asgr1*^–/–^ mouse liver were similar to those of WT counterparts, and the significantly altered genes were very limited (Figure 4A). Gene set enrichment analyses (GSEAs) showed improved metabolic signaling and reduced cholesterol-related signaling in *Asgr1*^–/–^ mouse livers (Figure 4B). Further analysis of the differentially expressed genes showed that 181 genes were significantly changed (fold change > 1.5, *q* value < 0.05), among which 104 genes were downregulated and 77 genes were upregulated (Figure 4C). Those dysregulated genes were substantially enriched into the metabolic pathway, indicating that the physiological functions of ASGR1 were relevant to metabolism (Figure 4D). A total of 34 genes were found to be changed in the metabolic pathway, which were involved in lipid metabolism, oxidoreductase, carbohydrate metabolism, and kinase (Figure 4E). Among those genes with altered expression, 12 of them were associated with lipid metabolism, in which 11 genes were downregulated and 1 gene was upregulated, as shown in the heatmap. Overall, this finding indicates that ASGR1 may play a physiological role in the regulatory network of lipid homeostasis.

### ASGR1 deficiency leads to fewer processed nSREBPs in the nucleus.

**Figure 5:**
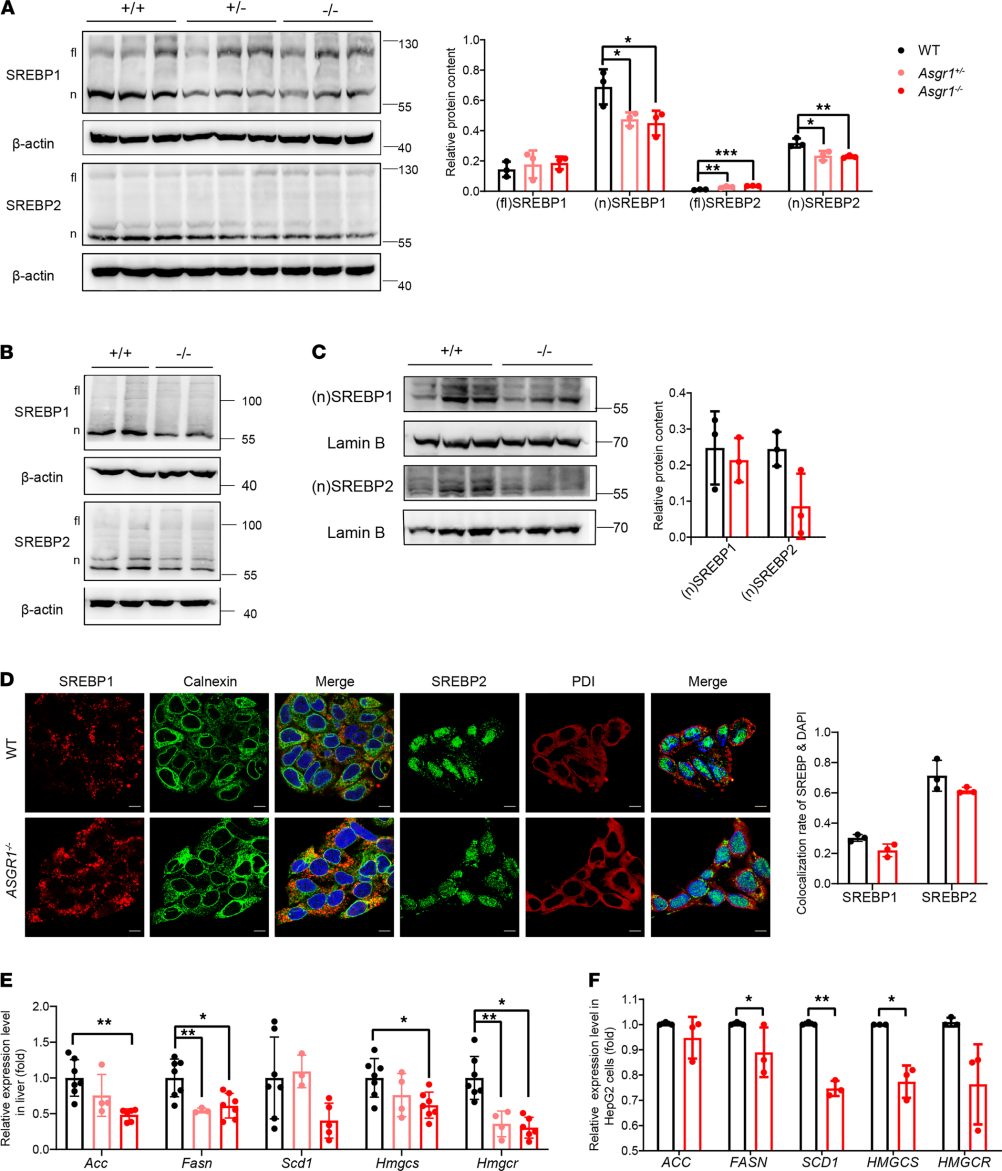
ASGR1 deficiency leads to fewer processed nSREBPs in the nucleus. In mice, (**A**) SREBP content was analyzed by Western blot in the whole protein of liver tissues, *n* = 3. fl, full length; n, nucleus. Parallel analyses were conducted on HepG2 cell lines. (**B**) SREBP content was analyzed by Western blot in the whole protein of HepG2 cell lysates. Representative images of immunoblotting shown, *n* = 3. Nucleic protein was isolated from HepG2 cell lysates, and (**C**) SREBP content in nucleus protein was analyzed by Western blot. SREBP location in cytoplasm and nucleus in hepatic cells was analyzed by immunofluorescence staining, *n* = 3. (**D**) Representative images to display SREBP location (scale bar: 10 μm). Calnexin and PDI are markers for ER. Colocalization rate of SREBP and DAPI shown on the right. Each point represents 1 experimental replicate, *n* = 3. (**E**) The mRNA expression level of SREBP-targeted genes in mouse livers. WT, *n* = 7; *Asgr1*^+/–^, *n* = 4; *Asgr1*^–/–^, *n* = 7. (**F**) The mRNA expression level of SREBP-targeted genes in HepG2 cells, *n* = 3. All data are shown as means ± SD. *P < 0.05, **P < 0.01, ***P < 0.001 as compared with the indicated WT by 1-way ANOVA among 3 groups and by unpaired, 2-tailed *t* test between 2 groups.

SREBPs are transcriptional factors that regulate the genes for lipid synthesis, transportation, and metabolism in multiple tissues. Because cholesterol and fatty acid metabolism showed substantial changes, we hypothesized that phenotypes of ASGR1 were related to SREBP family proteins. By detecting SREBP1 and SREBP2, we found that the protein content of nSREBP1 and nSREBP2 was lower in ASGR1-deficient mouse livers and HepG2 cells, while the content of full-length proteins was increased in mice, indicating that the activation was inhibited (Figure 5, A and B). Next, we separated nuclear and cytoplasmic proteins from HepG2 cells and confirmed that the level of nSREBP1/2 was reduced in the nuclei of *ASGR1*^–/–^ cells, while more full-length SREBP1/2 was accumulated in the cytoplasm (Figure 5C and Supplemental Figure 5A). The immunofluorescence data further indicated that less nSREBP1/2 was distributed in the nuclei of ASGR1^–/–^ cells (Figure 5D). To assess the SREBP signaling activity, the mRNA expression of its targeted genes in lipogenesis (*ACC*, *FASN*, and *SCD1*) and cholesterol synthesis (*HMGCS* and *HMGCR*) was analyzed in mouse livers and HepG2 cells. All of those inspected genes were transcriptionally downregulated, indicating that lipogenesis was inhibited in the absence of ASGR1 (Figure 5, E and F); in addition, the expression of *MTTP* and *PCSK9* related to LDL assembling and endocytosis was also significantly decreased (Figure 2, C and F; and Figure 3, B and E). These data suggested that ASGR1 deficiency might lead to defects in SREBPs’ activation and signaling.

### Higher INSIG1 is responsible for the ER retention of SREBPs in ASGR1 deficiency.

Cholesterol and protein chaperones are known to affect the activation of SREBPs. To clarify the factor responsible for SREBPs’ regulation, cholesterol level in mouse liver was measured. Fed with a normal diet without cholesterol addition, *Asgr1*^–/–^ mice’s cholesterol content in livers was significantly lower than that of WT mice (Figure 1H). In addition, bile acid content in the liver and feces, as well as the expressions of bile acid synthesis-related genes in liver, had no significant difference among mice with or without ASGR (Supplemental Figure 3, D–F).

**Figure 6:**
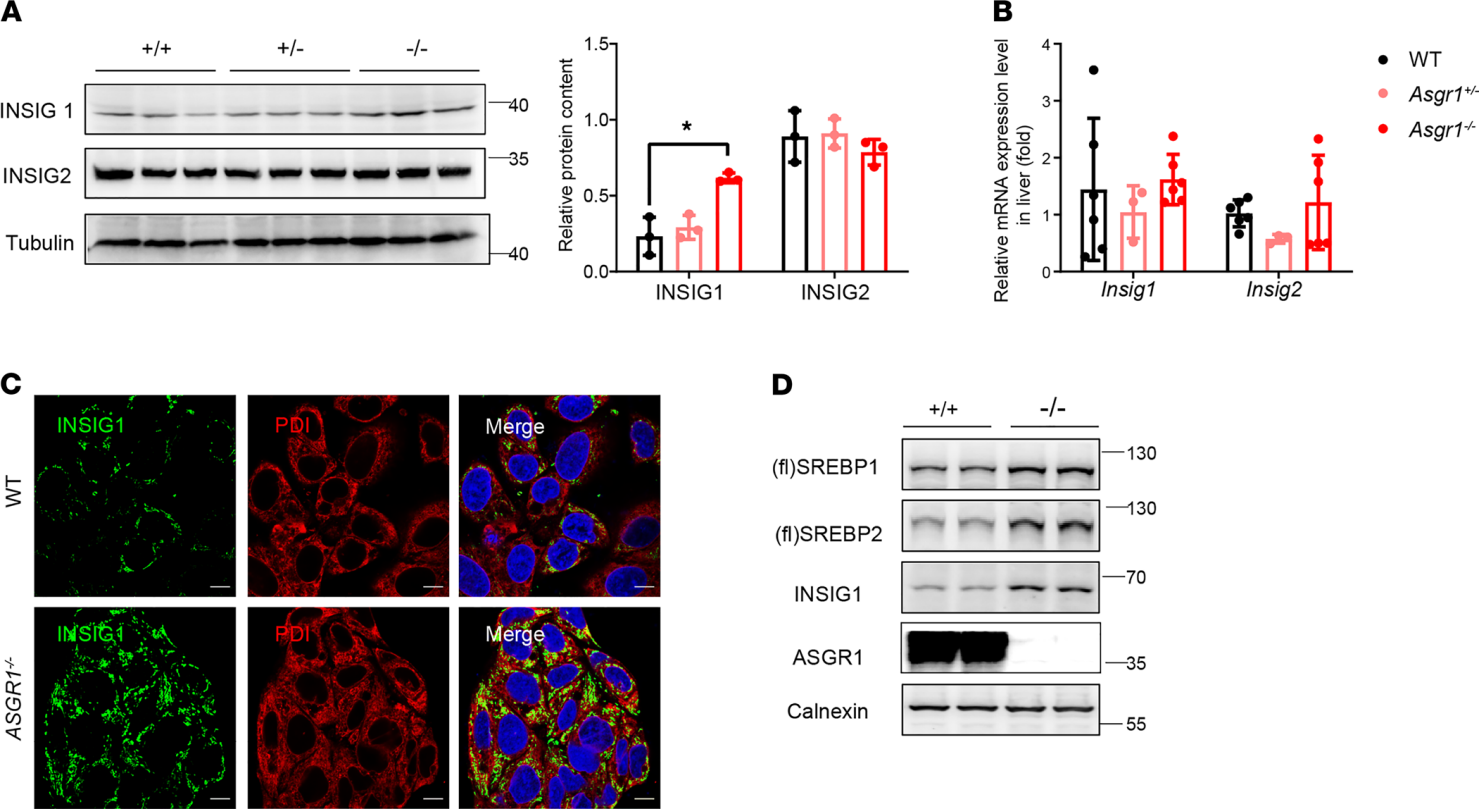
High INSIG1 level is responsible for the ER retention of SREBPs in ASGR1 deficiency. In mice, (**A**) INSIG1 and INSIG2 protein contents were analyzed by Western blots in liver tissues, *n* = 3. (**B**) mRNA expression level of *Insig1* and *Insig2* in liver tissues (WT, *n* = 6; *Asgr1*^+/–^, *n* = 3; *Asgr1*^–/–^, *n* = 6). In HepG2 cells, (**C**) representative images of immunofluorescence staining for INSIG1 and ER marker PDI (scale bar: 10 μm). (**D**) SREBP and INSIG1 protein contents of ER fraction isolated from HepG2 cells were analyzed by Western blots. Representative images of the immunoblotting shown, *n* = 3. All data are shown as the means ± SD. *P < 0.05, as compared with the indicated WT by 1-way ANOVA among 3 groups. Statistical significance between 2 groups was assessed with an unpaired, 2-tailed *t* test.

SREBPs were transported from the ER to Golgi apparatus for proteolytic double cleavages and activation. To demonstrate whether there were defects of the SREBP activation process under ASGR1 deficiency, all of those proteins involved in the process were screened by Western blot analysis. We found that only INSIG1 was increased in ASGR1-knockout mice, which might contribute to the retention of SREBPs in the ER (Figure 6A and Supplemental Figure 5B). However, the mRNA level of *Insig1* and *Insig2* showed no significant changes among mice (Figure 6B). Immunofluorescence staining of INSIG1 in HepG2 cells showed that the level of INSIG1 was markedly increased and predominantly located on the ER of *ASGR1*^−/−^-HepG2 cells (Figure 6C). Furthermore, Western blot analysis, which was performed specifically on ER proteins, showed that the full-length SREBP1/2 and INSIG1 were predominantly accumulated in the ER of ASGR1-deficient cells (Figure 6D). In summary, these results suggest that the increased INSIG1 protein might be responsible for the ER retention of SREBPs in ASGR1 deficiency.

### INSIG1 knockdown reverses the lipid profile and SREBP signaling in ASGR1 deficiency.

**Figure 7:**
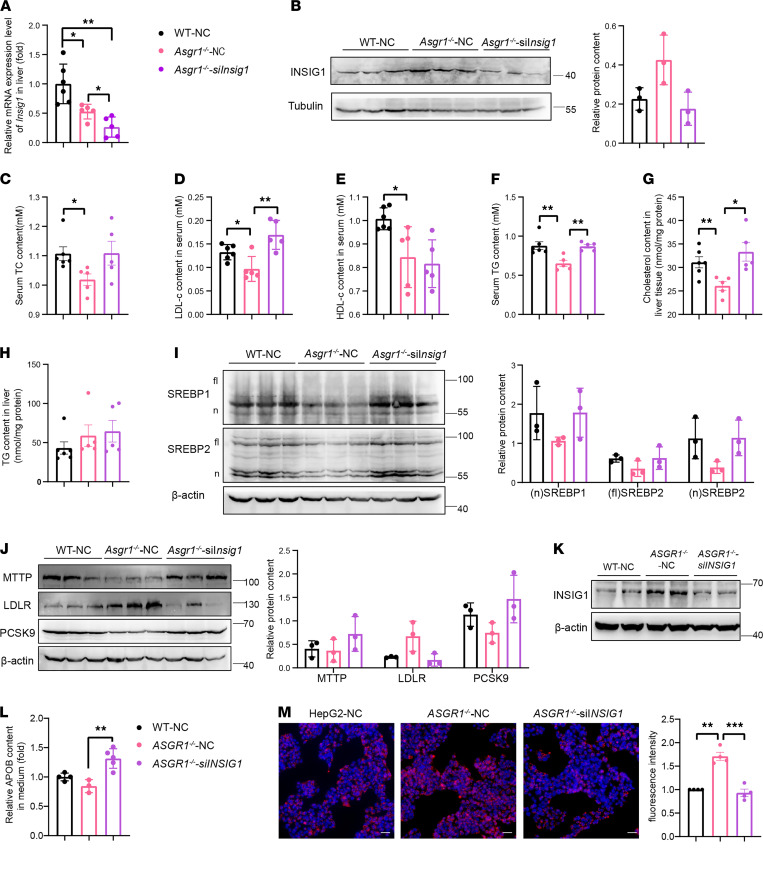
*INSIG1* knockdown reversed the lipid profile and SREBP signaling in ASGR1 deficiency. INSIG1-knockdown assays were conducted in vivo and in vitro via siRNA. Specific siRNA-*INSIG1* (si*INSIG1*) and siRNA-negative control (NC) sequences for mice or humans were applied for the assay, respectively. In mice, after the injection of siRNA, (**A**) mRNA expression level of Insig1 in liver was checked by RT-PCR, *n* = 5. (**B**) The corresponding INSIG1 protein in liver tissues was analyzed by Western blots, and gray intensity of each band relative to Tubulin was shown, *n* = 3. Mice were fasted overnight. Biochemical index of (**C**–**F**) TC, LDL-c, HDL-c,and TG in serum; (**G** and **H**) cholesterol; and TG were checked (WT, *n* = 6; *Asgr1*^+/–^, *n* = 5; *Asgr1*^–/–^, *n* = 5). (**I** and **J**) SREBP1, SREBP2, MTTP, LDLR, and PCSK9 protein content in liver tissues and gray intensity of each band relative to β-actin shown, *n* = 3. In HepG2 cells, (**K**) INSIG1 protein content after transfection with siRNA was analyzed by Western blots, *n* = 3. (**L**) Secreted APOB content in the cell medium was analyzed by ELISA (WT-NC, *n* = 4; ASGR1^–/–^-NC, *n* = 3; ASGR1^–/–^-si*INSIG1*, *n* = 5). The LDL uptake assays were conducted in transfected cells. (**M**) Representative images of LDL-dil accumulation (scale bar: 20 μm) and fluorescence intensity quantitative analysis, *n* = 4. All data are shown as the means ± SD. *P < 0.05, **P < 0.01, ***P < 0.001, as compared with the indicated WT by unpaired, 2-tailed *t* test between 2 groups.

To test whether the increased INSIG1 is responsible for ASGR1 deficiency–associated lipid phenotypes, we performed INSIG1-knockdown experiments mediated by 2*′*OMe+5*′*chlo modified siRNA in ASGR1-deficient mice (*Asgr1*^–/–^-si*Insig1*). Whereas knockdown of *Insig1* mRNA resulted in the reversal of the increased INSIG1 protein levels to near the WT levels (Figure 7, A and B), the phenotypic features were reversed in *Asgr1*^–/–^ mice as manifested by decreased serum TC, LDL-c, and TG and liver cholesterol content (Figure 7, C, D, F, and G), while there were no obvious changes in serum HDL-c and liver TG content (Figure 7, E and H). Furthermore, while the protein level of INSIG1 in *Asgr1*^–/–^ mice was reduced to the level similar to the one in WT mice, the reduction of nSREBP1/2 proteins was also reversed, and the scramble siRNA groups (WT-NC and *Asgr1*^–/–^-NC) had no marked effects on lipid profile or on nSREBP levels (Figure 7I). Likewise, the protein levels of SREBP-targeted genes, *MTTP*, *LDLR*, and *PCSK9*, displayed reversed states (Figure 7J).

In parallel, the same *INSIG1*-knockdown assay was applied in HepG2 cells (Figure 7K). Then phenotypic features were reversed with 40% increase of APOB secretion and LDL uptake down to the level of WT (Figure 7, L and M). Overall, these results indicated that the reduction of processed nSREBPs was primarily caused by increased INSIG1 under ASGR1-deficient conditions.

### Restoring ASGR1 expression rescues INSIG1-mediated network defects.

**Figure 8:**
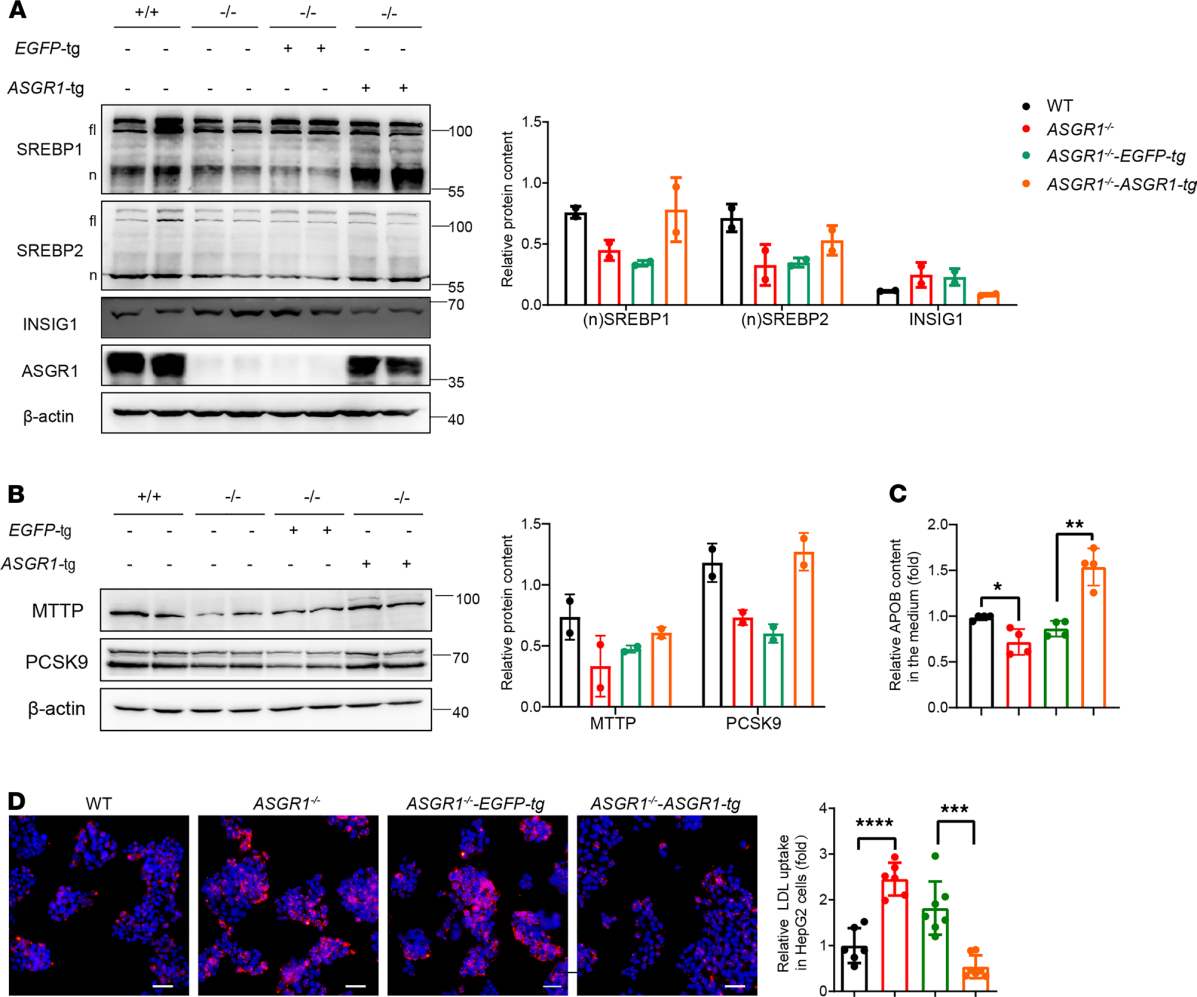
Restoring ASGR1 expression rescues INSIG1-mediated network defects. *ASGR1* overexpression assays were conducted in *ASGR1*^–/–^ HepG2 cell *ASGR1* overexpression (*ASGR1*^–/–^-*ASGR1*-tg) cells and control group with *EGFP* overexpression (*ASGR1*^–/–^-*EGFP*-tg) were generated for following assays. (**A**) SREBP and INSIG1 protein content analysis by Western blots. Representative images of the immunoblotting and gray intensity of each band relative to β-actin shown, *n* = 3. (**B**) MTTP and PCSK9 were analyzed by Western blots. Representative images of the immunoblotting and gray intensity of each band relative to β-actin was shown, *n* = 3. (**C**) Secreted APOB measurement by ELISA, *n* = 4. (**D**) The LDL uptake assays. Representative images of LDL-dil accumulation (scale bar: 20 μm) and fluorescence intensity quantitative analysis shown, *n* = 3. All data are shown as the means ± SD. *P < 0.05, **P < 0.01, ***P < 0.001, ****P < 0.0001 as compared with the indicated WT by unpaired, 2-tailed *t* test between 2 groups.

To confirm the cause-effect linkage between ASGR1 and the SREBP-mediated events, the *ASGR1* expression was restored by transfection into *ASGR1*^−/−^-HepG2 cells (*ASGR1*^−/−^-*ASGR1*-tg). Compared with the control cells (*ASGR1*^−/−^-*EGFP*-tg), nSREBP1/2 were increased, while INSIG1 was decreased (Figure 8A). Consequently, the expression levels of MTTP and PCSK9 were also increased in *ASGR1*^−/−^*-ASGR1*-tg cells (Figure 8B). Correspondingly, the related phenotype of APOB secretion increased by 50%, and LDL uptake returned to the WT level in *ASGR1*^−/−^*-ASGR1*-tg cells (Figure 8, C and D). Overall, these results indicate that restoration of ASGR1 expression in ASGR1-null cells rescued higher INSIG1-mediated SREBP network defects and the related phenotypes.

## Discussion

Homeostasis takes a great body of components to form a systematically coordinated network for dynamic adjustments. Generally, the network contains at least 3 key players, including the sensors, effectors, and a regulatory center. For regulation of cholesterol homeostasis, one of the sensors is a cholesterol-binding domain distributed in many proteins (including *trans* factors) that regulate cholesterol production, transportation, and metabolism. In addition, a DNA *cis* element (the SRE) is embedded in the regulatory region of many genes that encode proteins serving as effectors to regulate cholesterol homeostasis. The control center is a transcriptional factor family, i.e., the SREBPs. In this complicated network, we have demonstrated that ASGR1 substantially participates in the regulatory network of serum lipid homeostasis through INSIG1-mediated SREBP signaling. The hypomorphic effect of ASGR1 results in a healthy and favorable lipid profile, which is primarily generated from the differential turnover of the plasma lipoproteins. In a dose-dependent manner, hypomorphic manipulations led to more LDLR on cell membrane and less assembled VLDL in hepatocytes, resulting in more LDL uptake and less VLDL secretion in cell and mouse models, which represented a substantially lower serum lipid content. This phenotype closely mimics the feature found in those human carriers with ASGR1 mutations.

The first report of ASGR1 mutation was based on a population genetic study and found a 12 bp deletion in ASGR1, which led to a loss of function. The heterozygous mutation carriers had a lower level of non–HDL-c and TG, and the ASGR1 haploinsufficiency was associated with a lower risk of CAD (3). Moreover, a recent study on 104 patients revealed that the expression level of ASGR1 in peripheral blood monocytes was positively correlated with serum TC and LDL-c levels at ages younger than 60 (26). Previously, *Asgr1*-knockout mice were established by replacing exons 2–3, which contain the ATG translational initiation codon and transmembrane domain (27). These ASGR1-knockout mice were unable to clear asialofetuin and asialo-orosomucoid. However, the measurements of LDL and VLDL revealed a decreased trend with large standard deviations without a statistical difference, and the serum TC and TG contents also had no significant difference. The failure to observe significant changes of lipid profile may be due to conditions including those particular knockout mouse lines, limited analyzed group samples, and the sex-influenced variation. In our *Asgr1*-knockout mice, all of the 9 exons of *Asgr1* on the genome had been deleted. The phenotypic plasma lipid profile of ASGR1-deficient mice was consistent with the observation in human *ASGR1* mutation carriers, and a decrease on hepatic cholesterol and TG content was also observed, which further confirmed that the ASGR1 haploinsufficiency effect on lipid homeostasis resulted in a healthy lipid profile. The *Asgr1*-knockout mice showed normal growth and development, and transcriptome analysis suggested that *Asgr1* knockout only led to decreased expression of a group of genes related to lipid metabolism. Those findings suggest that the inhibition of ASGR1 function might be a new strategy for the development of lipid-lowering drugs.

The mechanistic study revealed that the hypomorphic phenotype of ASGR1 was predominantly caused by reduced production of nSREBPs. Reduced nSREBP production seems to explain most of the phenotypes observed under ASGR1 deficiency conditions, except for the increased INSIG1 protein. SREBP family proteins are transcriptional factors for various genes, which play a pivotal role in lipid homeostasis (17, 18). Both nSREBP1 and nSREBP2 were decreased in ASGR1 deficiencies, which led to the physiological benefits of the plasma lipid profile. Genes involved in lipogenesis and lipoprotein metabolism, particularly the expression of 2 SREBP-targeted genes, *MTTP* and *PCSK9*, were markedly inhibited under ASGR1-deficient conditions. VLDL/LDL particles mediated lipid secretion from the liver, and there are 2 steps generally involved in VLDL/LDL assembly. First, the MTTP transports the lipids onto APOB to form precursor particles. Then, the precursor particles and TG droplets combine to form mature VLDL/LDL (28). MTTP is transcriptionally regulated by SREBPs, and the lack of MTTP results in APOB cotranslational degradation (29–31). We observed that the MTTP was reduced along with lower levels of APOB, when ASGR1 was deficient, resulting in decreased VLDL/LDL secretion.

LDLR recycling plays a critical role in the endocytosis of LDL, and hepatic uptake of serum LDL-c facilitates serum lipid homeostasis (32). *Ldlr* mRNA decreased in the liver of ASGR1-deficient mice because of decreased nSREBPs. By contrast, the LDLR protein levels markedly increased 1.5-fold in the liver tissues of *Asgr1*^−/−^ mice. The high level of LDLR is independent of its transcriptional regulation. Therefore, protein stability and degradation of LDLR should be considered causes of increased LDLR in the ASGR1-deficient condition. It was speculated that ASGR1 might regulate lipid homeostasis via interacting with LDLR. Our data from colocalization and co-IP assays indicated that there was no direct interaction between ASGR1 and LDLR. Another SREBP-targeted gene, *Pcsk9*, was reduced by 50% in ASGR1-deficient hepatocytes. The PCSK9 protein is a key mediator for LDLR degradation by ubiquitination, and its inhibitors have been applied to clinical trials for hypocholesterolemia (33, 34). *LDLR* and *PCSK9* are downstream genes of SREBPs. While nSREBPs were decreased, the mRNA of both genes declined. In contrast, increased LDLR protein was revealed in all ASGR1-deficient conditions. It seems plausible that the SREBP suppression is responsible for the higher LDLR level in ASGR1 deficiency. The gene expression of PCSK9 is negatively regulated by SREBPs, and PCSK9 protein is a key mediator for LDLR internalization and degradation by ubiquitination. It is fair to speculate that the reduced PCSK9 is accountable for less degradation of LDLR. To confirm this judgment, we conducted a rescue experiment through overexpression of PCSK9 in HepG2 and HepG2-ASGR1^–/–^ cells and found out that the LDLR levels were reduced while the PCSK9 protein levels were increased either in HepG2 or in HepG2-ASGR1^–/–^ cells. However, there was no significant difference between the 2 groups (Supplemental Figure 4D). Collectively, the suppression of SREBP accounted for low PCSK9 and less degradation of LDLR that may result in higher LDLR level and LDL endocytosis.

Functions of SREBPs depend on their activation by double proteolytic cleavage. During the process, the transportation of SREBPs from the ER to the Golgi and to the nucleus is the key event that is involved with many factors for trafficking and cleavage. We screened all the known related proteins and only identified INSIG1 to be increased (Figure 6). To test whether the increased INSIG1 is responsible for ASGR1 deficiency–associated lipid phenotypes and SREBP suppression, we performed knockdown or restored expression of key factors in the ASGR1-deficient mice and cells and found that INSIG1 knockdown or ASGR1 restoration reversed the ASGR1 deficiency–associated lipid profile and SREBP suppression. This suggests that there is a signaling axis, ASGR1/INSIG1/SREBP, in which INSIG1 is the primary change factor and independent of SREBP under ASGR1 deficiency, and hypomorphic ASGR1 modulates lipid homeostasis via INSIG1-mediated SREBP signaling suppression.

INSIG1 is a protein promoting ER retention of SREBPs, while it is the targeted gene that exerts negative feedback control of SREBPs (35, 36). However, the higher INSIG1 protein is independent of the SREBP signaling suppression in the ASGR1-deficient condition. Thus, it seems that other mechanisms might play a major role in the increased INSIG1 in ASGR1 deficiency. INSIG1 is ubiquitinated and degraded by GP78 when oxysterols are depleted, while oxysterols stabilize it by binding within its transmembrane domains (37, 38). Although uptake of LDL-c was increased, the hepatic cholesterol was decreased in ASGR1-depleted mice, indicating that sterols were not the critical factor in inhibiting SREBPs’ translocation in our model. Moreover, *Insig1* is remarkably induced by insulin (39, 40). Notably, desialylation of the insulin receptor (IR) induces its activation and insulin signal transduction (41, 42). It is worthwhile to further investigate whether ASGR1 mediates IR degradation and regulates the expression of *Insig1*.

Overall, our observation clearly demonstrated that the increased INSIG1 was a primary change factor and responsible for the ASGR1 deficiency–associated lipid profile changes and nSREBP suppression. More importantly, the higher INSIG1 protein was independent of the SREBP signaling suppression in the ASGR1-deficient condition. The deep mechanistic roots of the ASGR/INSIG1 linkage are worth clarifying and uncover potential targets for cholesterol-lowing drug and dyslipidemia treatment development.

## Methods

### Mice

*Asgr1*-knockout mice were generated at Cyagen. All mouse lines were of C57BL/6 genetic background or their offspring. Mice aged about 10–13 weeks were used for experiments, and the age difference of the mice was no more than 1 week for the same experiment. Mice were housed in a controlled environment (12-hour daylight cycle), with free access to normal food and water.

*Asgr1*-knockout mice on the C57BL/6 background were generated using CRISPR/Cas9. Briefly, Cas9 mRNA and a pair of single-guide RNAs were mixed and injected into zygotes. Genome-edited F0 *Asgr1^+/–^* mice (Cyagen) were bred with C57BL/6 mice for 2 rounds to dilute the off-target effects. WT and *Asgr1^–/–^* littermates were generated by breeding *Asgr1^+/–^* mice. The detailed schematic diagrams of generating *Asgr1*-knockout mice models are shown in Supplemental Figure 1. Sequences of single-guide RNAs are shown in Supplemental Table 1.

### Cell line and cell culture

Cells were maintained at 37°C with humidified air and 5% CO_2_. HepG2 and H1 cell lines were gifts from Pan Guangjin (Guangzhou Institutes of Biomedicine and Health, Chinese Academy of Sciences, Guangzhou, China). HepG2 cells were cultured with DMEM (low glucose; Gibco) supplemented with 10% FBS (Gibco) and 1% nonessential amino acid (NEAA, Gibco). Cells were changed to be cultured in DMEM without FBS overnight before use. CRISPR/Cas9-induced gene edition in human hepatoma HepG2 cells was established according to a previous study (43). For targeting, 0.8 million HepG2 cells were transfected with 4 μg Cas9-G418 plasmid and 1 μg *ASGR1* single-guide RNA (sgRNA) plasmid for each sgRNA by electrotransfection with nucleus kits (Lonza). Then, the cells were seeded onto Matrigel-coated, 24-well plates. Targeted cells were screened by supplementing with 50 μg/mL G418 in cultured medium for 48 hours after 24 hours’ recovery. Thereafter, cells were replated to 96-well plates for single-cell culture. Clones were collected for sequencing and expansion. PCR products were used for sequencing identification, and primers for PCR amplification are shown in Supplemental Table 1.

### Serum and hepatic lipid assays

Blood was collected from heart after fasting overnight, and plasma was further isolated via centrifugation at 970*g* for 10 minutes at 4°C. TC, LDL-c, HDL-c, and TG levels were measured using an automatic biochemical detector (Guangzhou Laboratory Animal Monitoring Institute). Snap-frozen liver samples were weighed and homogenized in 20 volumes of ice-cold lysis buffer. The lysate was then centrifuged at 431*g* for 5 minutes at 4°C, and the supernatant was transferred to a new tube. A total of 20 μL of supernatant was used for protein level detection, and the remaining supernatant was heated in a metal bath for 10 minutes at 70°C for further detection. Cholesterol and TG content were determined by enzyme kits from PPLYGEN, according to the manufacturer’s instructions.

### FPLC assay

Pooled plasma samples from mice fasted overnight were subjected to FPLC. A total of 700 μL mixed plasma from 7 mice per group was filtrated through a 0.22 μm filter (MilliporeSigma). Then 300 μL plasma was injected onto a Superose 6 10/300 GL column (GE Healthcare BioSciences AB) and eluted at a constant flow rate of 0.4 mL/min PBS. Fractions of 600 μL were collected and assayed for cholesterol and TG. For apolipoprotein analysis, 10 μL of each FPLC fraction was subjected to SDS-PAGE and immunoblotting against APOB or APOA.

### Oral lipid tolerance and hepatic lipid secretion assays

For oral lipid tolerance assay, mice were fasted overnight followed by tail vein injection of 15% Triton WR1399 in saline (500 mg/kg body weight) (MilliporeSigma). Then, 200 μL olive oil was intragastrically administrated into mice. Blood samples were collected at 0, 1, 2, 3, and 4 hours after WR1399 injection for further lipid content detection. For hepatic lipid secretion assay, mice were fasted for 5 hours. Blood from the tail vein was collected for further detection. Mice were injected with Triton WR1399 via tail vein. Blood was collected 1, 2, and 3 hours after WR1399 was administrated for further lipid content detection.

### APOB secretion assay in vitro

HepG2 cells were seeded 1 day before the assay. Then cells were rinsed twice with PBS before culturing for 12 hours in a serum-free medium. The amount of secreted APOB in medium was measured using APOB ELISA kit (Abcam) according to the instructions.

### Isolation of primary hepatocyte

Primary hepatocytes were isolated from chow diet–fed WT, Asgr1^+/^, and Asgr1^–/–^ mice by 2-step collagenase (MilliporeSigma) perfusion digestion and low-speed centrifugation (50*g*, 4°C), then cultured in the collagen-coated dish with DMEM (high glucose, Hyclone) containing 10% FBS (Gibco), 1% NEAA, 1% ITS (Gibco), 1% penicillin and streptomycin (Life Technologies), and 0.1 μM dexamethasone (Selleck). All cells were serum-starved for 12 hours before harvest.

### LDL uptake assay in vitro

HepG2, primary hepatocytes and H1-derived hepatocytes were serum-starved for 12 hours before incubating with 10 μg/mL LDL-dil (Invitrogen) for 3 hours. Hoechst 33258 was used to stain the nucleus before analysis with a fluorescence microscope (Axiovert A1, Zeiss). The intensity of LDL-dil was normalized by number of cells. To quantify the fluorescence intensity values and calculate an average, at least 5 representative images were selected to be analyzed. Experiments were repeated 3 times individually at least. All the intensity of dye was quantified by ImageJ (NIH).

### RNA-Seq assay and analysis

Male mice, WT and *Asgr1*^–/–^, were fasted overnight before collection of the liver tissues. Total RNA of each sample was extracted using TRIzol Reagent/RNeasy Mini Kit (QIAGEN). Total RNA of each sample was quantified and qualified by Agilent 2100/2200 Bioanalyzer (Agilent Technologies), with NanoDrop (Thermo Fisher Scientific). A total of 1 μg RNA was used for the following library preparation. Next-generation sequencing library preparations were constructed according to the manufacturer’s protocol. The sequences were processed and analyzed by GENEWIZ. All the sequencing data have been submitted to the National Center for Biotechnology Information’s Gene Expression Omnibus data bank and the access number is GSE178370.

### Immunofluorescence

HepG2 cells were fixed by 4% paraformaldehyde for 10 minutes at room temperature after being washed by PBS twice. Then cells were blocked and permeabilized with 0.1% Triton X-100 (MP Biomedicals) and 10% FBS diluted in PBS for 30 minutes at room temperature. After that, cells were incubated with primary antibody SREBP1 (Thermo Fisher Scientific, 1:100), SREBP2 (Abcam, 1:100), INSIG1 (Proteintech, 1:200), CALNEXIN (Enzo, 1:200), and PDI (MilliporeSigma, 1:200) incubated at 4°C overnight. After 5 washes with PBS, cells were incubated with Alexa Fluor 488 goat anti-rabbit IgG (H+L) and Alexa Fluor 568 goat anti-mouse IgG (H+L) secondary antibody (Life Technologies) for 1 hour at room temperature with gentle shaking. At last, the samples were washed with PBS 3 times before staining the nucleus with DAPI for 5 minutes. Immunofluorescence images were obtained and analyzed with Zeiss 710 NLO confocal microscopy.

For fluorescence intensity quantification, ImageJ was applied, and relative intensity was quantified by intensity divided by view area. For colocalization, the rate was quantified using ImageJ with colocalization finder. For each sample, no less than 5 representative images were analyzed to quantify the fluorescence intensity values and calculate an average. Experiments were repeated 3 times individually at least.

### Preparation of nuclear extracts and ER fractions

Nucleic and cytoplasmic proteins were obtained by nucleic protein and cytoplasmic protein extraction kit (Beyotime) according to instructions, then used for further analysis by immunoblotting. About 2 × 10^7^ cells were collected after 2 washes by precooled PBS, then centrifuged at 500*g* for 5 minutes at 4°C. Then the ER proteins were extracted according to the Endoplasmic Reticulum Isolation Kit (BestBio) instructions. The obtained proteins were administrated with RIPA buffer (Beyotime), then heated at 100°C for 10 minutes with 4× loading buffer before being subjected to SDS-PAGE.

### Knockdown assay

#### Insig1-knockdown assay in vivo with siRNA.

The siRNA for mice was modified with cholesterol conjugation and methylation for higher efficiency. AML12 cells (Cell Bank of Chinese Academy of Sciences) were used to screen the best siRNA sequences with high knockdown efficiency from 3 candidate sequences according to the instructions for siRNA transfection in cells. The selected *Insig1* siRNA and control were dissolved in 200 μL saline solution before injection. Mice about 12 weeks old were injected with 2.5 nmol siRNA per mouse via caudal vein. The mice were injected 3 days for 3 times in a total of 9 days and then were sacrificed for experiments. All the *Insig1* siRNA sequences and the controls were from Ribo Company. The sequence information was listed in Supplemental Table 1.

#### Transfection of siRNA in cells.

HepG2 cells were prepared for transfection of *INSIG1* siRNA. *INSIG1* siRNA and control were preincubated with Lipofectamine 3000 (Invitrogen) for 15 minutes to form a complex according to the manufacturer’s instructions. The mixing complex was added to HepG2 cell medium to a final concentration of 50 nM. Then 72 hours after the transfection, the cells were collected, and the knockdown efficiency was analyzed by real-time quantitative PCR and Western blots. All the *INSIG1* siRNA sequences and the control were from IGE company. The sequence information was listed in Supplemental Table 1.

### Molecular cloning

The full length of *ASGR1* cDNA was obtained from HepG2 cells and cloned into a lentivirus vector, pRlenti. Virus was packed in 293T cells (Cell Bank of Chinese Academy of Sciences), and supernatant containing active virus was collected for infection. Positive cells were screened out by puromycin (MilliporeSigma) resistance in a dose of 1.2 μg/mL for HepG2. Primers for PCR or siRNA sequences are in Supplemental Table 1.

### Real-time quantitative PCR

Total RNA was isolated from liver tissue stored in liquid nitrogen by TRIzol (Med Chem Express). Real-time PCR was performed with Bio-Rad CFX96. The experimental procedure was carried out according to the reagent instructions. The relative mRNA expression was analyzed by normalizing with β-actin in all genes. The sequences of primers were summarized in Supplemental Table 1.

### Statistics

Statistical significance between 2 groups was assessed with an unpaired, 2-tailed *t* test using Microsoft Excel and among 3 or more was assessed with 1-way ANOVA. All data represent means ± SEM. Statistical significance is denoted by *P < 0.05, **P < 0.01, and ***P < 0.001.

### Study approval

All animal studies, including mouse feeding and operation experiments, were performed in accordance with the experimental animal ethics committee of Guangzhou Institutes of Biomedicine and Health.

## Author contributions

YXL conceived and supervised the project. YX, JT, and XY contributed equally to this work. YXL, YX, and JT prepared the manuscript. YX and JT designed experiments, performed most of the cell experiments, and analyzed the data. The method used in assigning the authorship order among co–first authors is the importance of each author’s work. YW and XY performed most of the animal experiments. YC and KY performed the primary hepatocyte separation experiments. TP and YZ performed the embryonic stem cell hepatic differentiation experiments. JZ, F Yuan, and F Yang assisted with the routine experiments. XL supported the statistical analysis. AG edited the manuscript.

## Supplementary Material

Supplemental data

## Version 1 10/08/2021

Electronic publication
