# Supplementary material for: Hypomorphic ASGR1 modulates lipid homeostasis via INSIG1-mediated SREBP signaling suppression
Source: JCI Insight. 2021 Oct 8;6(19):e147038. doi: 10.1172/jci.insight.147038 (PMC8525641; doi:10.1172/jci.insight.147038)
Supplement: Supplemental data [file jciinsight-6-147038-s039.pdf]

Supplemental figures

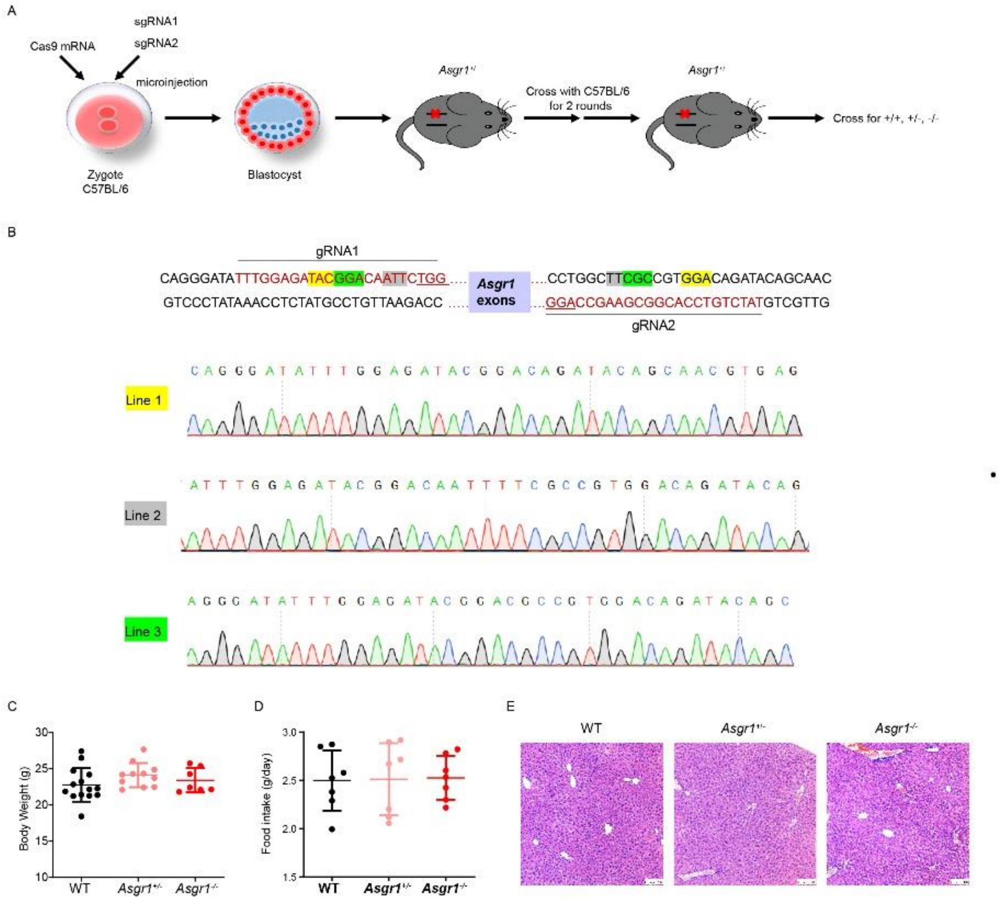

Figure S1 Generation and characterization of ASGR1-deficient mice models.

(A) Schematic diagram for generation of *Asgr1* knockout mice using CRISPR/Cas9.

(B) DNA sequencing showing CRISPR-induced deletion of *Asgr1* in genome.

(C) Body weight of WT (n=14), *Asgr1*$^{+/-}$ (n=11) and *Asgr1*$^{-/-}$ (n=7) male mice.

(D) Food intake of WT, *Asgr1*$^{+/-}$ and *Asgr1*$^{-/-}$ male mice per day on average, n=7.

(E) Representative H&E staining images of mice liver paraffin sections, scale bars, 100 μ m.

All data are shown as the means ± SD.

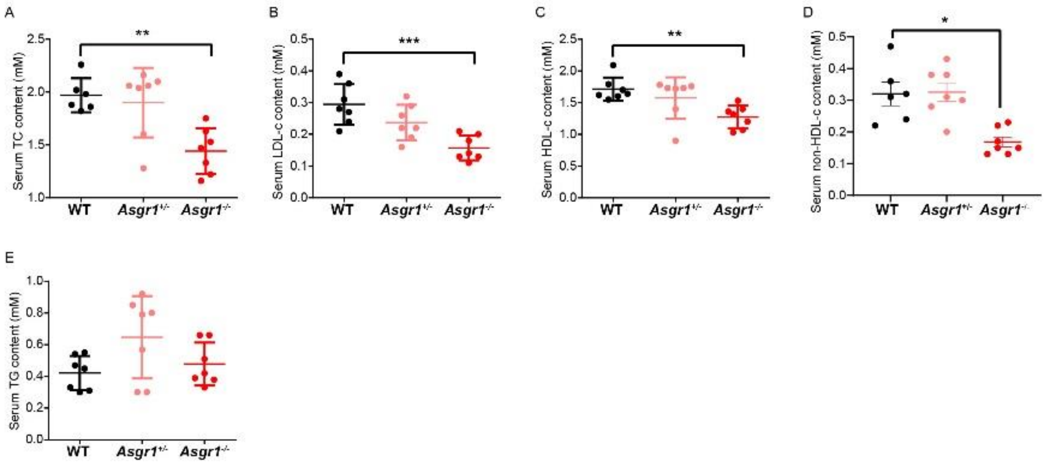

Figure S2 Characterization of ASGR1-deficient female mice

Female mice at age about 10-13 weeks were fasted overnight.

(A) Total cholesterol (TC), (B) LDL-c, (C) HDL-c, (D) non-HDL-c and (E) TG content in serum of female mice were detected by automatic biochemical analyzer, n=7.

All data are shown as the means ± SD. *p<0.05, **p < 0.01, ***p < 0.001 as compared to the indicated WT by one-way ANOVA.

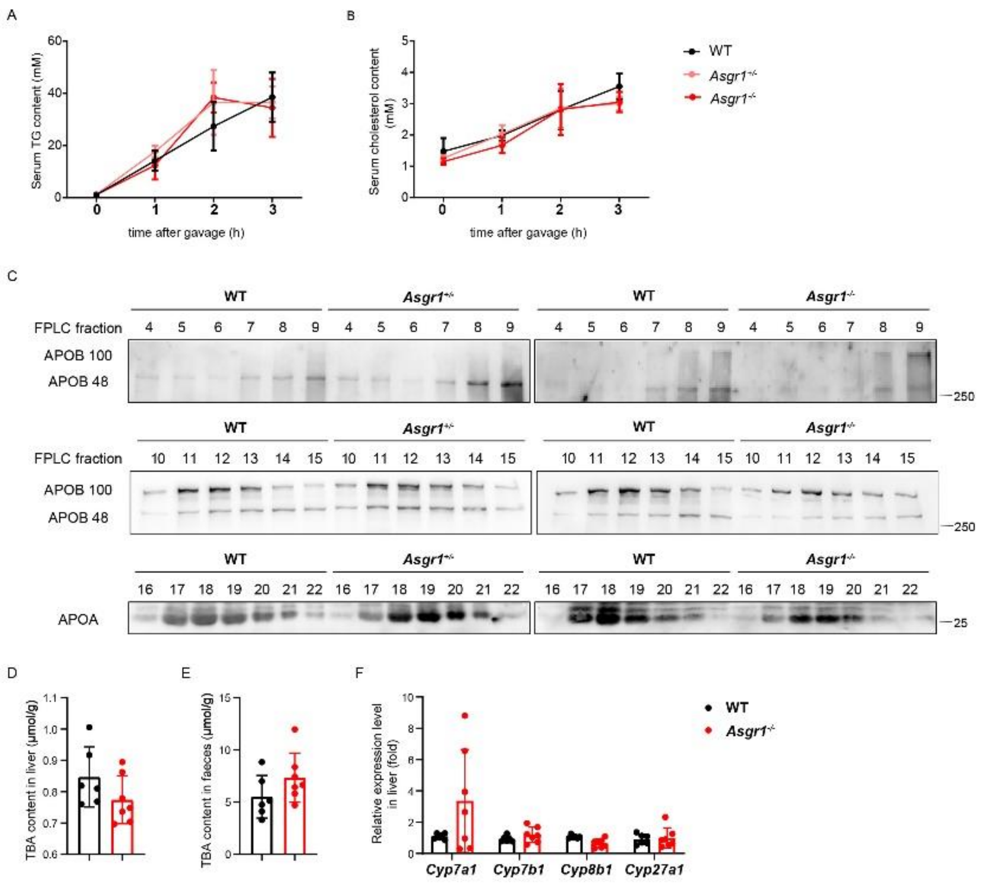

Figure S3 Metabolic characterization of ASGR1 deficient mice.

Male mice at age about 10-13 weeks were used for assays.

(A, B) Lipid tolerance assays for WT, *Asgr1*$^{+/-}$, *Asgr1*$^{-/-}$ mice. Plasma TG and cholesterol content following i.p. injection with tyloxapol (500mg/kg body weight) after oral olive oil gavage (200 μl per mouse) in 5-hr-fasted mice (n=5 per group). (A) TG content of plasma at each time point. (B) Cholesterol content of plasma at each time point.

(C) Western blot of APOB100, APOB48 and APOA in eluted fractions from FPLC fractions containing CM, LDL or HDL particles respectively (n=5 per group).

(D) Total bile acid (TBA) content in mice liver were detected (WT, n=6; *Asgr1*$^{-/-}$, n=7).

(E) TBA content in faces were detected (WT, n=6; *Asgr1*$^{-/-}$, n=7).

(F) Relative mRNA expression level of bile acid synthesis related genes in mice liver (WT, n=6; *Asgr1*$^{-/-}$, n=7).

All data are shown as the means ± SD.

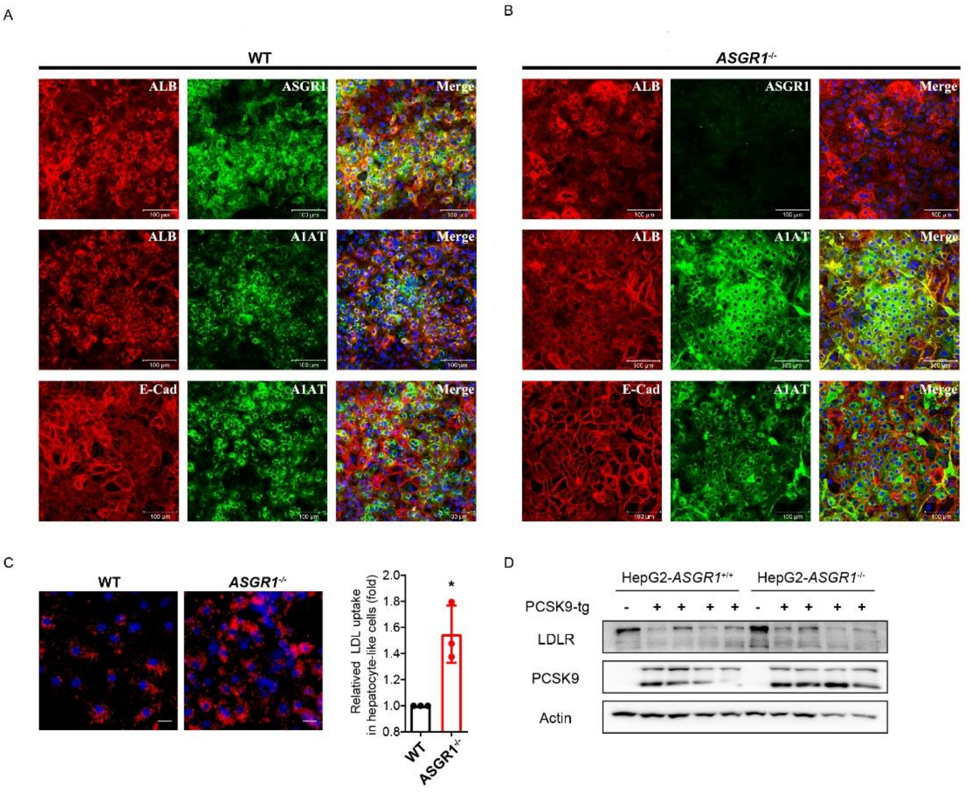

Figure S4 ASGR1 deficiency increases LDL endocytosis in hepatocyte-like cells.

Human embryonic stem cells H1 were differentiated to mature hepatocyte-like cells.

(A, B) Immunofluorescence of mature hepatocytes marker proteins, Albumin (ALB), A1AT, E-cadherin (E-cad) and ASGR1 in hepatocyte-like cells derived from H1 and H1-ASGR1$^{-/-}$, n=3. Scale bars, 100μm.

(C) LDL uptake in hepatocyte-like cells. Cells were starved overnight, and incubated with LDL-dil for 3h. Representative images of LDL-dil accumulation in cells were showed, scale bar 20μm. The florescent intensity was shown on the right. Each point represents one of the experimental replicates, n=3.

(D) LDLR protein content changed as PCSK9 protein content changed in HepG2 cells with/ without ASGR1, n=3.

All data are shown as the means ± SD. *$p < 0.05$ as compared to the indicated WT by Students t test.

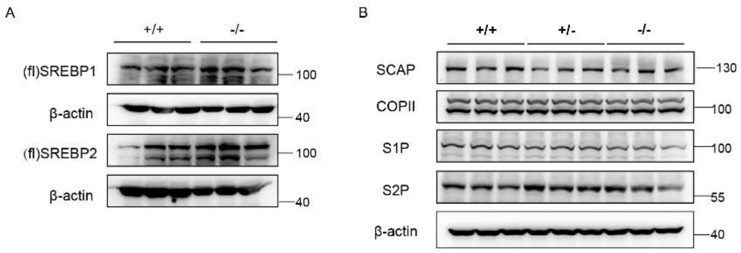

Figure S5 Expression level of SREBPs and related protein

(A) Cytoplasm protein of HepG2 cells were separated and detected SREBP1/2 protein by western blotting, n=3.

(B) Western blotting of SCAP, COPⅡ， S1P and S2P in mice liver tissues, n=3.

Supplemental tables

REAGENT	SOURCE	IDENTIFIER
**Antibody**		
Rabbit polyclonal anti-ASGR1	Proteintech	11739-1-AP
Rabbit polyclonal anti-MTTP antibody	Affinity	DF6591
Rabbit polyclonal anti-APOB antibody	Proteintech	20578-1-AP
Rabbit Polyclonal anti-APOA1 antibody	Proteintech	14427-1-AP
Rabbit polyclonal anti-Anti-PCSK9 [EPR7627(2)]	Abcam	ab181142
Rabbit polyclonal anti-LDL Receptor [EP1553Y]	Abcam	ab52818
mouse monoclonal anti-SREBP1antibody(2A4)	Thermo Fisher	MA5-11685
Rabbit polyclonal anti-SREBP2 antibody	Abcam	ab30682
mouse Monoclonalanti-PDI Antibody	thermo Fisher	MA3-019
Rabbit polyclonal anti-Calnexin antibody	Enzo	ADI-SPA-860
Rabbit polyclonal anti-INSIG1 antibody	Proteintech	22115-1-AP
Rabbit polyclonal anti-INSIG2 antibody	Proteintech	24766-1-AP
Rabbit polyclonal anti-SCAP antibody	Proteintech	12266-1-AP
Rabbit polyclonal anti-S1P antibody	Abcam	ab140592
Rabbit polyclonal anti-S2P antibody	Abcam	ab140594
Rabbit polyclonal anti-COPII antibody	Thermo Fisher	PA1069A
Rabbit polyclonal anti-Tubulin antibody	Cell Signaling Technology (CST)	2146
Rabbit monoclonal anti-βactin antibody	Cell Signaling Technology (CST)	4970
Rabbit monoclonal anti-LaminB1(D4Q4Z)	Cell Signaling Technology (CST)	12585S
Goat anti-mouse	KangCheng Bio	KC-MM-

IgG(H&L)[HRP]		035(1ml)
Goat anti-Rabbit IgG(H&L)[HRP]	KangCheng Bio	KC-RB-035(1ml)
Donkey anti-mouse IgG 488	Life	A21202
Donkey anti-mouse IgG 568	Life	A10037
Donkey anti-Rabbit IgG 488	Life	A21206
Donkey anti-Rabbit IgG 568	Life	A10042
DAPI	Sigma	D9542
**Chemicals, Peptides, Recombinant Proteins, metarials and Assay kit**		
DMEM	Gibco	10567-022
Fetal Bovine Serum	Gibco	A115-500
NEAA	Gibco	11140-050
Penicillin-Streptomycin	Gibco	15140122
0.25% Trypsin-EDTA	Gibco	25200-114
ITS	Gibco	41400045
SuperoseTM 6 10/300 GL	GE	17-5172-01
Tissue and cell triglyceride (TG) assay kit	APPLYGEN	E1013
Tissue total cholesterol enzymatic determination	APPLYGEN	E1016
Tyloxapol	SIGMA	T0307-10g
Human APOB ELISA kit	Abcam	ab108807
LDL-Dil	invitrogen	l3482
Nuclear protein and cytoplasmic protein extraction kit	Beyotime	P0028
Endoplasmic reticulum extraction kit	BestBio	BB-3605-2
Trizol	MCE	N/A
SYBR-green	Bio-RAD	N/A
Protease Inhibitor Cocktail Tablets	Roche	11 873 580 001
PHOS-STOP	Roche	04 906 837 001
PMSF	Beyotime	ST506
PVDF membrane	Merck Millipore	IPVH00010
Triton X-100	MP	N/A
Tris Base	Genestar	N/A
Agarose	Genestar	VA10252
**Cell lines and mice strains**		
Human: HepG2	N/A	
Human:293T	N/A	

Mouse: C57BL/6	Beijing Vital River Laboratory Animal Technology	
Mouse: C57BL/6 *Asgr1*$^{-/-}$	Cyagen	
**Quantitative RT-PCR primers**		
Primer name	Sequence	
Human-ARG1-forward	GTGGAAACTTGCATGGACAAC	
Human-ARG1-reverse	AATCCTGGCACATCGGGAATC	
Human-ASL-forward	CAGTGGACCCCATCATGGAGA	
Human-ASL-reveres	CAGTGGACCCCATCATGGAGA	
Human-CPS1-forward	AATGAGGTGGGCTTAAAGCAAG	
Human-CPS1-reverse	AGTTCCACTCCACAGTTCAGA	
Human-CYP2D6-forward	CCAACGGTCTCTTGGACAAAG	
Human-CYP2D6-reverse	GGGTCGTCGTACTCGAAGC	
Human-CYP3A4-forward	AAGTCGCCTCGAAGATACACA	
Human-CYP3A4-reverse	AAGGAGAGAACACTGCTCGTG	
Human-CYP2C9-forward	GCCTGAAACCCATAGTGGTG	
Human-CYP2C9-reverse	GGGGCTGCTCAAAATCTTGATG	
Human-CYP1A2-forward	ATGCTCAGCCTCGTGAAGAAC	
Human-CYP1A2-reverse	GTTAGGCAGGTAGCGAAGGAT	
Human-A1AT-forward	GGAGGCTCAGATCCATGAAGG	
Human-A1AT-reverse	GGTGTCCCCGAAGTTGACAG	
Human-GAPDH-forward	CAAAGTTGTCATGGATGACC	
Human-GAPDH-reverse	CCATGGAGAAGGCTGGGG	
Human-Ki67-forward	GCCTGCTCGACCCTACAGA	
Human-Ki67-reverse	GCTTGTCAACTGCGGTTGC	
Human-CK19-forward	GAACCATGAGGAGGAAATCAG	
Human-CK19-reverse	CATGTCACTCAGGATCTTGG	
Human-ALB-forward	TGGCACAATGAAGTGGGTAA	
Human-ALB-reverse	CTGAGCAAAGGCAATCAACA	
Human-HNF4α-forward	TGTACTCCTGCAGATTTAGCC	
Human-HNF4α-reverse	CTGTCCTCATAGCTTGACCT	
Human-AFP-forward	AGAACCTGTCACAAGCTGTG	
Human-AFP-reverse	GACAGCAAGCTGAGGATGTC	
Huamn-βactin forward	CTCCTCCTGAGCGCAAGTACTC	
Huamn-βactin reverse	TCCTGCTTGCTGATCCACATC	
Huamn-APOB forward	ACACACTGGACGCTAAGAGGA	
Huamn-APOB reverse	ACTTGTGCTACCATCCCATACT	
Huamn-MTTP forward	TTCATGCAAAATAGCGAGGTCT	
Huamn-MTTP reverse	GCTTCGGTTGTCTTCAGCTCTA	
Huamn-LDLR forward	TCTGCAACATGGCTAGAGACT	
Huamn-LDLR reverse	TCCAAGCATTCGTTGGTCCC	
Huamn-PCSK9 forward	CCTGGAGCGGATTACCCCT	

Huamn-PCSK9 reverse	CTGTATGCTGGTGTCTAGGAGA	
Huamn-SREBF1 forward	GCCCCTGTAACGACCACTG	
Huamn-SREBF1 reverse	CAGCGAGTCTGCCTTGATG	
Huamn-SREBF2 forward	CCTGGGAGACATCGACGAGAT	
Huamn-SREBF2 reverse	TGAATGACCGTTGCACTGAAG	
Huamn-ACC forward	ATGTCTGGCTTGCACCTAGTA	
Huamn-ACC reverse	CCCCAAAGCGAGTAACAAATTCT	
Huamn-FASN forward	CCGAGACACTCGTGGGCTA	
Huamn-FASN reverse	CTTCAGCAGGACATTGATGCC	
Huamn-SCD1 forward	TTCCTACCTGCAAGTTCTACACC	
Huamn-SCD1 reverse	CCGAGCTTTGTAAGAGCGGT	
Huamn-HMGCS forward	GATGTGGGAATTGTTGCCCTT	
Huamn-HMGCS reverse	ATTGTCTCTGTTCCAACTTCCAG	
Huamn-HMGCR forward	TGATTGACCTTTCCAGAGCAAG	
Huamn-HMGCR reverse	CTAAAATTGCCATTCCACGAGC	
Huamn-ASGR1 forward	GAGAGAGACGTTCAGCAACTTC	
Huamn-ASGR1 reverse	GGGACTCTAGCGACTTCATCTT	
Mouse-βactin forward	GGCTGTATTCCCCTCCATCG	
Mouse-βactin reverse	CCAGTTGGTAACAATGCCATGT	
Mouse-Apob forward	GCTCAACTCAGGTTACCGTGA	
Mouse-Apob reverse	AGGGTGTACTGGCAAGTTTGG	
Mouse-Mttp forward	AATGCGGGTCAACAGAGAGG	
Mouse-Mttp reverse	CTGGCTCGTTTTCATAGGAGTAG	
Mouse-Ldlr forward	TCAGACGAACAAGGCTGTCC	
Mouse-Ldlr reverse	CCATCTAGGCAATCTCGGTCTC	
Mouse-Pcsk9 forward	GCCCATCGGGAGATTGAGG	
Mouse-Pcsk9 reverse	TTCCCTTGACAGTTGAGCACA	
Mouse-Srebf1 forward	GCAGCCACCATCTAGCCTG	
Mouse-Srebf1 reverse	CAGCAGTGAGTCTGCCTTGAT	
Mouse-Srebf2 forward	CCAAGCTGGGCGATGGAT	
Mouse-Srebf2 reverse	TAGCATCTCGTCGATGTCCC	
Mouse-Acc forward	CTCCCGATTCATAATTGGGTCTG	
Mouse-Acc reverse	TCGACCTTGTTTTACTAGGTGC	
Mouse-Fasn forward	GGAGGTGGTGATAGCCGGTAT	
Mouse-Fasn reverse	TGGGTAATCCATAGAGCCCAG	
Mouse-Scd1 forward	TTCTTGCGATACACTCTGGTGC	
Mouse-Scd1 reverse	CGGGATTGAATGTTCTTGTCGT	
Mouse-Hmgcs forward	TGGCTATAAAGCTGCGGAGG	
Mouse-Hmgcs reverse	GGTGAAAGAGCCAAAGGGGA	
Mouse-Hmgcr forward	CGGGAGCCCTAGAGAACAAG	
Mouse-Hmgcr reverse	CTTGGATCCCACGCGGA	
**Oligonucleotides**		

Mice Asgr1 knock-out sgRNA sequence	gRNA1: TTTGGAGATACGGACAATTCTGG	
	gRNA2: TATCTGTCCACGGCGAAGCCAGG	
Mice ASGR1 knock-out Identification primers	Forward: TTTGAGAAGCCCTTGTCTTCCAGAG	
	Revers: ATGTGTCTGTGATCCTGAGCTGGGTA	
	Wt/He-Forward: GGAACAGACTACGAGACAGGCTTCCA	
Human ASGR1 knock-out sgRNA sequence	gRNA-1: GTATCAAGACCTTCAGCATCTGG	
	gRNA-2: GACCACCATCAGCTCAGAAAAGG	
Human ASGR1 knock-out Identification primers	Forward: TGATGGAGCCGTCAATCAGATG	
	Reverse: ACTGTCGGCTTCCTATGACG	
INSIG1-siRNA sequence for human	Forward: GACUGUGGAUUAUGACAAAdTdT	
	Reverse: UUUGUCAUAAUCCACAGUCdTdT	
Negative control-siRNA sequence for human	Forward: UUCUCCGAACGUGUCACGUdTdT	
	Reverse: ACGUGACACGUUCGGAGAAdTdT	
Insig1-siRNA sequence for mouse	Forward: CCACGCCAGTGCCAAATTA	
	Reverse: TAATTTGGCTCUGGCGUGG	
Negative control-siRNA sequence for mouse	Forward: provided by Ribobio	
	Reverse: provided by Ribobio	
**Bacterial and Virus Strains**		
Cas9-G418 plasmid	Gift from Professor Lai Liangxue	
gRNA plasmid	Gift from Professor Lai Liangxue	
pRlenti plasmid	Gift from Professor Miguel Angel Esteban	
